# CtMYB1 regulates flavonoid biosynthesis in safflower flower by binding the CAACCA elements

**DOI:** 10.1371/journal.pone.0337921

**Published:** 2025-12-10

**Authors:** Yanxun Zhou, Jie Wang, Yanni Peng, Chao Chen, Bin Xian, Ziqing Xi, Chaoxiang Ren, Jin Pei, Jiang Chen

**Affiliations:** 1 State Key Laboratory of Southwestern Chinese Medicine Resources, Chengdu University of Traditional Chinese Medicine, Chengdu, China; 2 College of Pharmacy, Chengdu University of Traditional Chinese Medicine, Chengdu, China; University of Delhi, INDIA

## Abstract

**Background:**

Safflower (*Carthamus tinctorius* L.) is a valuable herb known for its flowers, which are rich in flavonoids and are used for promoting blood circulation and preventing atherosclerosis. However, the molecular regulation of flavonoid biosynthesis in safflower is still poorly understood. In this study, we identified a *AtMYB12* homologous gene, *CtMYB1*, in safflower and characterized its sequence. The flower protoplast transient expression system and virus-induced gene silence (VIGS) technique were established in safflower and we tested the role of *CtMYB1* in the regulation of flavonoid biosynthesis. The correlation between CtMYB1 and flavonoid synthesis genes was analyzed using a molecular interaction assay.

**Results:**

Flower protoplasts transient expression showed that flavonoid biosynthesis genes *CtC4H2*, *CtF3H4*, and *CtHCT12* were upregulated after transfection with *CtMYB1*. Meanwhile, VIGS showed that the transfected petals were lighter in color, and there was a decrease in the amount of the major component Hydroxysafflor yellow A (HSYA) compared to the control. Yeast one-hybrid experiments demonstrate that CtMYB1 can bind to the promoters of flavonoid biosynthesis genes (*CtC4H2*, *CtF3H4*). The interaction analysis by the use of Biacore system revealed that CtMYB1 binds to the CAACCA element of flavonoid biosynthesis genes promoters.

**Conclusions:**

CtMYB1 regulates flavonoid biosynthesis in safflower flower by binding the CAACCA elements of flavonoid biosynthesis related genes promoters, which sheds light on the molecular regulation of flavonoid biosynthesis in safflower. The flower protoplasts expression system and the VIGS system established in safflower are valuable tools for studying the function of genes involved in the regulation and biosynthesis of active compounds in safflower.

## Introduction

The dried tubular flowers of safflower (*Carthamus tinctorius* L.), named HONG HUA, are widly used in traditional medicine, due to its efficacy in promoting blood circulation, removing blood stasis and relieving pain. Flavonoids, including Hydroxysafflor yellow A(HSYA), naringenin, kaempferol, and quercetin, are the primary active components of safflower flowers. These flavonoids have been demonstrated to enhance blood circulation and prevent atherosclerosis. The composition and content of flavonoids directly influence the quality of safflower [1,2]. In recent years, with the development of traditional Chinese medicine, the demand for safflower has risen. Despite the continuous expansion of the safflower planting area, the issue of the scarcity of flavonoid compounds in safflower remains unresolved. Thus, increasing the content of flavonoid compounds in safflower and cultivating and screening new safflower strains with high flavonoid content are of great importance. Transcription factors play a crucial regulatory role in the synthesis of plant secondary metabolites. Relevant literature indicates that transcription factors associated with flavonoid biosynthesis mainly include MYB, bHLH, WD40, bZIP, etc. Based on our research group’s previous studies, this research focuses on the MYB transcription factors in safflower [3,4].

The MYB transcription factor family is known to play a key role in the regulation of flavonoid biosynthesis in plants such as *Arabidopsis thaliana*, where the sixth family of MYB transcription factors is primarily involved [5]. The *AtMYB12* gene, as well as its homologues *AtMYB11* and *AtMYB111*, has been shown to regulate flavonoid biosynthesis in *Arabidopsis* [6,7]. However, currently, reports regarding MYB transcription factors involved in flavonoid biosynthesis in safflower mainly focus on screening and cloning, and there are few research reports on their specific regulatory mechanisms. The function of genes can be verified through stable or transient expression systems [8,9]. The transient expression system, especially the protoplasts expression system, offers several advantages. It enables rapid, cost-effective, and high-throughput analysis, along with high transformation efficiency [10]. Protoplasts expression systems have been established in a wide range of plants, such as *Arabidopsis* [11], cucumber [12], Rice [13], and other plants. Meanwhile, the Virus-induced gene silencing (VIGS) technique, which is also a type of transient expression system based on the tobacco rattle virus (TRV), is widely used for verifying plant gene functions, for example, in tobacco [14]. Based on the principle of surface plasmon resonance, Biacore can detect the interactions of various molecules, like proteins and nucleic acids, in real-time without labeling. It also provides comprehensive interaction information, including binding specificity, affinity, kinetics, and competition.

In this study, we cloned and characterized a *AtMYB12* orthologue, *CtMYB1*, in safflower. Using the safflower flower protoplasts transient expression system and VIGS technique, we investigated the role of *CtMYB1* in the regulation of flavonoid biosynthesis. Our results shed light on the molecular regulation of flavonoid biosynthesis in safflower. The establishment of flower protoplasts expression system and VIGS technique in safflower are a valuable tool for studying gene function, particularly those involved in the regulation and biosynthesis of active compounds of safflower.

## Materials and methods

### Total RNA extraction and the cloning of *CtMYB1*

Safflower was grown at the Medicinal Botanical Garden of Chengdu University of Traditional Chinese Medicine. Total RNA was extracted using TRIzol reagent (TIANGEN, China). The purity of the obtained RNA was determined using a NanoPhotometerTM (N60 Touch) (IMPLEN, Germany). The first-strand cDNA was synthesized from total RNA using a cDNA Reverse Transcription Kit (Takara, Beijing, China). A mixed cDNA library was created from roots, stems, leaves, and flowers. We analyzed the conserved domains of MYB transcription factors, and based on the conserved amino acid sequences, a pair of degenerate primers (5’-GRBTDMGRAARGGTKCWTGGA-3’ [Forward], 5’-GCWATHARDGACCAYCTRTT-3’ [Reverse]) was designed and used to amplify the *MYB* core sequence from safflower using Polymerase Chain Reaction (PCR) (S1 Table). The PCR protocol consisted of an initial denaturation step at 94°C for 5 minutes, followed by 35 cycles of denaturation at 94°C for 30 seconds, annealing at 42°C for 30 seconds, extension at 72°C for 1 minute, and a final extension step at 72°C for 7 minutes. The PCR product was cloned into a *pMD19-T* vector (Takara, Beijing, China) and sequenced by Tsingke (Chengdu, China). The 3’ and 5’ end sequences of the core sequence were cloned using Rapid Amplification of cDNA Ends (RACE), and the full-length cDNA of *CtMYB1* was finally cloned.

### Construction of *CtMYB1* recombinant vectors

To express the gene *CtMYB1* in safflower flower protoplasts, the plasmid *pET-32a* (+)*-CtMYB1* was constructed. The PCR product of the gene *CtMYB1* was cloned into the plasmid *pMD19-T* (Takara, Beijing, China) following the manufacturer’s instructions. Primers were designed specifically for this purpose: the forward primer 5’-GCCATGGCTGATATCGGATCCATGATCCAAGATCAAGATC-3’ and the reverse primer 5’-ACGGAGCTCGAATTCGGATCCTTAATTAGTCACATTATAT-3’ (S2 Table), both of which contain the BamH I site (underlined).The *pMD19-T-CtMYB1* plasmid and *pET-32a (+)* were digested with the restriction enzyme BamH I (also from Takara, Beijing, China) and joined together using T4 DNA ligase(Takara, Beijing, China), resulting in the correct recombinant expression plasmid, which was named as *pET-32a (+)-CtMYB1* (S2 Table).

The *CtMYB1* fragment was inserted into *pA7-YFP* (Yellow Fluorescent Protein), replacing the *YFP*. High Fidelity PCR SuperMix Ⅱ (manufactured by TransGen Biotech, located in Beijing, China) was used to amplify the plasmid under the following conditions: 98°C for 3 minutes, followed by 34 cycles of 98°C for 30 seconds, 62°C for 30 seconds, 72°C for 90 seconds, and a final extension of 72°C for 5 minutes. The primers 5’-TTCCTGCAGCCCGGGGGATCCATGATCCAAGATCAAGATCA-3’ (forward) and 5’-ACTAGTATGGTGAGCGGATCCATTAGTCACATTATATATAC-3’ (reverse) (S2 Table), both containing the BamH I site (underlined), were used to construct the gene *pA7-CtMYB1-YFP*.To determine the subcellular localization of the CtMYB1 protein in safflower protoplasts, the fluorescence signal from YFP was detected using the Olympus FV1200 Confocal Laser Scanning Microscope (Olympus, Japan) with an excitation wavelength of 488 nm and 50% laser power.

*pTRV1* and *pTRV2* are the two RNA strands of the Tobacco rattle virus (TRV) vectors commonly used in virus-induced gene silencing (VIGS) technology. Moreover, *pTRV2* contains multiple cloning sites. To silence the *CtMYB1* gene in safflower petals, the plasmid *pTRV2-CtMYB1* was constructed. Primers containing the EcoRⅠ cleavage site (underlined) (Takara, Beijing, China) and approximately 15 bp of the *pTRV2* sequence were designed according to the homology arm cloning method (S2 Table). Using these primers, the *CtMYB1* gene was cloned into a 489-base-pair fragment containing the *pTRV2* homology arm sequence. Then, the *CtMYB1* fragment with the homology arm was inserted into the EcoR I-digested *pTRV2* using Exnase II ligase. High-Fidelity PCR Super Mix II (TransGen Biotech, Beijing, China) was used to amplify the plasmid under the following conditions: pre-denaturation at 98°C for 3 minutes, followed by 34 cycles of denaturation at 98°C for 30 seconds, annealing at 58°C for 30 seconds, and extension at 72°C for 90 seconds, with a final extension at 72°C for 5 minutes. Finally, the plasmid *pTRV2-CtMYB1* was successfully constructed.

### The isolation of protoplasts in safflower

The stock solutions were prepared according to the formulas shown in Table 1. Protoplast transfection was performed based on Jiang Chen’s research on maize protoplasts [15] with some modifications. We carried out the isolation of flower protoplasts in safflower for the first time. The young flowers of safflower were cut into pieces with a side-length of approximately 0.5 mm using sharp razors and were pretreated for 10 minutes in a dish containing mannitol. The chopped pieces were then transferred to an enzyme hydrolysis solution [0.6 M mannitol, 10 mM MES, 10 mM CaCl2, 0.1% bovine serum albumin (BSA), 1.5% Cellulase RS (from Yakult, Japan) and 0.75% Macerozyme R-10 (from Yakult, Japan)]. The solution was incubated in the dark at 25°C with shaking at 60 rpm for 3–4 hours. Before adding the CaCl2 and BSA, the enzymes (Cellulase RS and Macerozyme R-10) were activated by thermal treatment at 55°C for 10 minutes. The chopped flower pieces were gently shaken three times during the incubation process to ensure complete enzymolysis. After incubation, an equal volume of precooled W5 solution (154 mM NaCl, 125 mM CaCl2, 5 mM KCl, 2 mM MES((2-(N-Morpholino) ethanesulfonic acid)), pH 5.8) was added to the enzyme hydrolysis solution, and the mixture was shaken at 80 rpm for 4 minutes to stop the hydrolysis. The mixture was then filtered through a 40 μm nylon sieve, and the residue was washed three times with W5 solution. The filtrate was transferred to a 50-mL centrifuge tube and horizontally centrifuged at 100 g for 3 minutes at room temperature using a Centrifuge Allegra X-30R (Beckman, USA) to collect the protoplasts. The supernatant was discarded, and the protoplasts were resuspended in 20 mL of precooled MMG solution (15 mM MgCl2, 0.4 M mannitol, 4 mM MES, pH 5.7). The solution was then horizontally centrifuged at 100 g for 1 minute, and the supernatant was discarded. The protoplasts were resuspended in 3 mL of precooled MMG and kept on ice for 30 minutes. The upper liquid was discarded to obtain high-quality protoplasts.

**Table 1:** Stock solution.

Reagents	Formulas
0.2 M MES	3.9 g of MES was dissolved in 100 mL and the pH was adjusted to 5.7 with KOH (of water)
0.8 M Mannitol	72.8 g Mannitol was dissolved in 500 mL(of water)
1 M CaCl_2_	14.7 g CaCl_2_·H_2_O was dissolved in 100 mL(of water)
2 M KCl	14.9 g KCl was dissolved in 100 mL(of water)
2 M MgCl_2_	20.3 g MgCl_2_ was dissolved in 50 mL(of water)
10% BSA	1 g BSA was dissolved in 10 mL(of water)

### The transformation of safflower petal protoplasts

The aim of this experiment was to investigate the effects of different centrifugal forces (200 g, 150 g, 100 g), different extraction sites, and different flowering times on the state of the prepared safflower protoplasts. We selected tubular flowers with the ovaries removed 2 days and 4 days after flowering. These flowers were divided into three different samples: whole flowers, corolla lobes, and corolla tubes. Each sample was cut into 0.5-millimeter segments for subsequent processing.

The transfection steps were as follows: First, 10 μg of *pA7-CtMYB1-YFP* plasmid DNA and positive plasmids(*pA7-YFP*, pro9, pro10, pro13, Pro14, pro15, pro16, pro17) were added to a 2-mL sterile centrifuge tube [16]. The plasmid DNA was evenly distributed at the bottom of the centrifuge tube by gently pipetting. Subsequently, 100 μL of suspended protoplasts was slowly added, and then the mixture was gently pipetted up and down several times to ensure thorough mixing. Immediately after that, 120 μL of PEG solution (40% PEG4000(Polyethylene Glycol), 0.8 M mannitol, 100 mM calcium chloride) was added, and the solution was gently pipetted up and down about 5–8 times again to achieve uniform mixing. The mixture was incubated at room temperature (around 25°C) for 20 minutes. During the incubation process, any shaking was avoided. After incubation, 460 μL of W5 solution was slowly added along the wall of the centrifuge tube, and the centrifuge tube was gently rotated to dilute the transfection mixture. Then, the solution was placed in a centrifuge and centrifuged horizontally at a centrifugal force of 100 g for 3 minutes. After centrifugation, the supernatant was carefully aspirated and discarded, and the protoplasts were resuspended in 1 mL of MMG solution. The protoplasts were also evenly dispersed by gentle pipetting. The MMG solution containing the protoplasts was incubated in the dark at room temperature for 12 hours. Finally, the incubated protoplasts were centrifuged horizontally at 200 g, 150 g, and 100 g for 3 minutes in sequence. The precipitated protoplasts were collected for subsequent experiments.

### Protoplasts RNA extraction and quantitative reverse transcription polymerase chain reaction (qRT-PCR) analysis

After transforming *pA7-CtMYB1-YFP* into safflower protoplasts, RNA was extracted from the protoplasts. Protoplasts without plasmid transformation were used as a blank control (CK), and RNA was also extracted from the blank control. We used the MicroElute Total RNA kit (Omega Bio-Tek, USA) to extract protoplast RNA. The purity of the RNA samples was assessed using a NanoDrop spectrophotometer (IMPLEN, Germany), and the A260/A280 ratios of all samples were found to be within the range of 1.9-2.1, indicating a pure RNA preparation. cDNA was synthesized from the purified RNA using the PrimeScriptTM RT reagent Kit with gDNA Eraser (Takara, Beijing, China) following the standard protocol provided by the manufacturer.

Expression levels of selected genes were determined by qRT-PCR after the over-expression of the *CtMYB1* gene, using a CFX96TM Real-time System (Bio-Rad, Hercules, CA, USA). Three replicate samples were analyzed for each reaction. Primers for genes related to flavonoid synthesis were designed using Primer Premier 5 software (S3 Table). The 25S rRNA gene of *Carthamus tinctorius* L. was used as the reference gene to normalize expression levels in each cDNA sample. The qRT-PCR reaction mixture consisted of 1 μL of each forward and reverse primer, 1 μL of cDNA, 7 μL of double-distilled water, and 10 μL of SYBR Green II (Takara, Beijing, China), in a total volume of 20 μL. To detect expression levels of five genes involved in flavonoid biosynthesis in safflower flowers, qRT-PCR conditions were as follows: an initial denaturation step at 95°C for 3 min, followed by 40 cycles of denaturation at 95°C for 10 s, annealing at 58.5°C for 30 s, extension at 72°C for 30 s, and a final extension step at 72°C for 5 min.

### Establishment of the VIGS system in safflower flowers

*pTRV1*, *pTRV2* and the recombinant plasmid *pTRV2-CtMYB1* were transformed into Agrobacterium *C58C1*(Tsingke Biotech Company, Chengdu, China). The transformed Agrobacterium was cultured in 5 mL of TY medium containing 100 µg/mL kanamycin and resuspended in infiltration solution (10 mmol/L 2-morpholinoethanesulfonic acid + 40 mg/L acetosyringone + 10 mmol/L magnesium chloride, pH 5.6) to an OD_600_ of 1.5 ~ 2.0, so that the densities of each group were similar. The infiltration solution of Agrobacterium containing *pTRV1* was mixed with the infiltration solutions of Agrobacterium containing *pTRV2* and *pTRV2-CtMYB1* respectively at a volume ratio of 1:1, and incubated at room temperature. Equal amounts of fresh safflower petals at the early flowering stage were immersed in different infiltration solutions for vacuum infiltration. The infiltrated petals were placed in sterile Petri dishes containing sterile water for incubation and observation. They were first incubated in darkness at 5 °C for 3 days, and then counted immediately after removal (recorded as day 0), and then incubated at 25 °C under light conditions suitable for normal safflower growth for 6 days, with the water changed daily to maintain the aseptic environment. Among them, the safflower infiltrated with Agrobacterium transformed with *pTRV2-CtMYB1* was the experimental group (*pTRV2-CtMYB1*), the safflower infiltrated with Agrobacterium transformed with the empty vector *pTRV2* was the control group (CK), and the safflower infiltrated with the sterile infiltration solution was the blank group (0). The cultivated safflower petal samples were frozen in liquid nitrogen and stored in a-80 °C refrigerator for subsequent experiments. GUS(β-Glucuronidase) staining was used to determine the infiltration status, and trypan blue staining was used to assess the viability of sterile-cultivated safflower.

### Determination of HSYA Content by High-Performance Liquid Chromatography (HPLC)

The content of Hydroxysafflor yellow A was determined according to the method in the Chinese Pharmacopoeia. After the safflower material was cultured, a portion of it was freeze-dried. The freeze-dried material was precisely weighed, and then the mixture was then ground using a milling machine, and the resulting powder was centrifuged at 9000 rpm for 5 minutes, causing the powder to settle on the bottom of the tube. Subsequently, about 1.25 ml of 25% methanol was added, maintaining the same concentration as specified in the Pharmacopoeia. The mixture was subjected to ultrasonic treatment (300W power and 50 kHz frequency) for 40 minutes and then cooled. Afterwards, the powder was centrifuged once again at 9000 rpm for 5 minutes, and the supernatant was filtered through a 0.45µm filter membrane into an injection vial. The resulting sample was then injected following the aforementioned liquid phase conditions. The injection column used was the Eclipse XDB-C18, measuring 4.6 × 250 mm with a 5um particle size. The mobile phases used were as follows: Phase A consisted of a 0.7% phosphoric acid solution, Phase B was composed of methanol, and Phase C of acetonitrile. The three phases were eluted in an isocratic manner using a ratio of 72:26:2 respectively. The detection wavelength employed was 403 nm, and the injection temperature was room temperature. A volume of 10 ul was injected for analysis.

### Recombinant protein expression and purification

The recombinant plasmid *pET-32a-CtMYB1* was transformed into E. coli BL21 (DE3) (Tsingke Biotech Company, Chengdu, China). The transformed bacteria were grown in 5 mL LB medium containing 100 μg/mL Ampicillin at 37°C with shaking at 200 rpm until the OD600 reached 0.35-0.45. The expression of the CtMYB1 fusion protein was then induced with 1 mM isopropyl-1-thio-β-D-galactopyranoside (IPTG) at 200 rpm, 16°C, for 12 hours. The bacteria were centrifuged at 4°C and 12,000 rpm for 5 minutes, and the supernatant was discarded. The cell pellets were resuspended in 1 mL of water and centrifuged again at 12,000 rpm for 5 minutes. The pellet was then dissolved in precooled 1 mL of Lysis Buffer (50 mM HEPES(4-(2-Hydroxyethyl)-1-piperazineethanesulfonic acid), 500 mM NaCl, 5 mM imidazole, pH 7.0).

The sample was subjected to SDS-PAGE (Sodium Dodecyl Sulfate-Polyacrylamide Gel Electrophoresis) analysis by adding 10 μL of 5 × SDS loading buffer (Solarbio, Beijing, China) and boiling for 10 minutes, followed by centrifugation at 12,000 rpm for 5 minutes. The resulting supernatant was loaded onto a Tris-glycine gel in the 4–20% range and visualized using Coomassie Brilliant Blue G-250 staining.

To purify the soluble form of *CtMYB1*, BL21 (DE3) cells harboring *pET-32a-CtMYB1* were grown in 1 L of LB medium with 100 μg/mL Ampicillin at 16°C and 200 rpm for 12 hours. The cells were collected by centrifugation at 4°C and 12,000 rpm for 10 minutes, and the supernatant was discarded. The cells were resuspended in 50 mL of water, centrifuged, and resuspended in 30 mL of Lysis Buffer. The cells were lysed using a sonicator (SCIENTZ, Ningbo, China) on ice for 15 minutes at 30% duty. The soluble and insoluble fractions were separated by centrifugation at 4°C and 12,000 rpm for 45 minutes. The supernatant was filtered using a 0.45 μm filter, and the cellular debris was resuspended in 10 mL of water. 1 mL of Ni-NTA(Nickel-Nitrilotriacetic acid) was added to the supernatant and incubated at 4°C and 50 rpm for 1 hour. The final processed supernatant was applied to a column and eluted with increasing concentrations of imidazole (40, 80, and 160 mM). The eluted fractions were saved in centrifuge tubes for SDS-PAGE analysis.

### Analysis of the interaction between transcription factor CtMYB1 and promoter elements

There are often some cis-acting sequences upstream or downstream of the promoter, which can be recognized by transcription factors, thereby regulating the activity of the promoter. The MYB elements in the promoters of 10 genes related to flavonoid synthesis were predicted online using the PlantCARE tool (S4 Table). The promoter fragments containing the MYB elements were synthesized by a company, and biotin was labeled at the 5’-end of one of the single-strands, while the complementary strand was not labeled, as shown in S5 Table.

The synthesized single-strands were annealed into double-strands. The method is as follows: Dilute the single-strand and its complementary strand with water to 100 μM respectively, dissolve and mix them thoroughly, then mix equal volumes of them and mix again thoroughly. Add 1/10 volume of 10-fold DNA oligonucleotide annealing buffer, mix well. Incubate the mixture at 95°C for 5 minutes, take it out and cool it at room temperature for 2 hours before use, or store the annealed product at-20°C.

The Surface Plasmon Resonance (SPR) technology used by the Biacore system is capable of detecting real-time protein-protein and protein-nucleic acid interactions. In this study, the CtMYB1 protein was produced by induction with 1 mM IPTG for 12 hours at 16°C. The Biacore system is user-friendly and has various applications, making it a useful research tool for detecting and measuring protein interactions [17–19].

The core elements (S5 Table) in the promoter that interact with MYB were analyzed online using PlantCARE. In the Biacore assay, one of the interactants is immobilized on a sensor chip surface while the other is flowed over the surface in solution. The immobilized interactant is referred to as the ligand and the flowing interactant as the analyte. In this study, the Biacore T200 was used to measure protein-nucleic acid interactions. The CtMYB1 protein was diluted to 50 μg/mL in sodium acetate solution (pH 4.0) and immobilized on a CM5 sensor chip via amine coupling to a density of 300 resonance units (RU). The immobilized CtMYB1 was then used to capture core elements from the flavonoids biosynthesis genes promoter sequences. This study referred to our previous research on the flavonoids biosynthesis genes in safflower [20]. The data were recorded at 25°C using a running buffer of PBS-T (10 mM sodium phosphate, 150 mM NaCl, 0.005% Tween-20, pH 7.4) and 2% DMSO (Dimethyl sulfoxide).

Six concentrations of synthetic promoters (0, 2, 4, 8, 16, 32 nM) were injected at a flow rate of 30 μL/min, with a 4-minute contact time and 5-minute dissociation time. A blank immobilization was performed on one of the sensor chips to correct the binding response.

### Yeast one-hybrid assay

The yeast one-hybrid assay mainly used the Lac system to detect the binding of CtMYB1 to genes related to flavonoid synthesis. *p178* vector which contains the *CYC1* core promoter and a reporter gene, the *LacZ* gene, was used to ligate promoters of genes related to flavonoid synthesis. In the experiment, we selected two promoters of flavonoid synthesis gene (*CtF3H4* and *CtC4H2*) and ligated them into the *p178* vector. The Infusion system was employed in the experiment to directly clone each gene promoter into the p178 vector digested with *Xho* I. Meanwhile, *CtMYB1* was ligated into the *pPC86* vector. Primers with restriction enzyme sites (*Sal* I and *Sac* I) were designed, and after digestion, *CtMYB1* was ligated into the *pPC86* vector that had also been digested with the same enzymes. The constructed vectors were co-transformed into *EGY48* yeast cells. The transformed yeast cells were spread on SD/–Ura–Trp medium and cultured at 28°C for 2 days. Positive clones were picked and subjected to expanded culture, and the bacterial solution was spread on SD/–Ura–Trp + X-a-gal medium. Colonies that appeared blue on the plate indicated the presence of an interaction. The primer sequences are listed in Table 2.

**Table 2:** Sequences for promoter cloning and yeast one-hybrid.

Name	Primer sequences
*pCtF3H4*	F: GGAACAGTAGCTTCTAGAGAT
	R: AGTCACTGCTCTTATGTACTC
*pCtC4H2*	F: TTCCGGTTCCGATCTTCGGAAA
	R: GGACCAGAGGGTGGTTTCAAT
*pPC86-CtMYB1*	F: CGCGTCGACATGATCCAAGATCAAGATC
*pPC86-CtMYB1*	R: GCCGAGCTCTTAATTAGTCACATTATAT
*178- pCtF3H4*	F: AGTTATTACCCTCGAGGGAACAGTAGCTTCTAGAGAT
*178- pCtF3H4*	R: GGCGGATCTGCTCGAGAGTCACTGCTCTTATGTACTC
*178- pCtC4H2*	F: AGTTATTACCCTCGAGTTCCGGTTCCGATCTTCGGAAA
*178- pCtC4H2*	R: GGCGGATCTGCTCGAGGGACCAGAGGGTGGTTTCAAT

## Results and discussions

### Cloning and sequence analysis of *CtMYB1*

**Fig 1:**
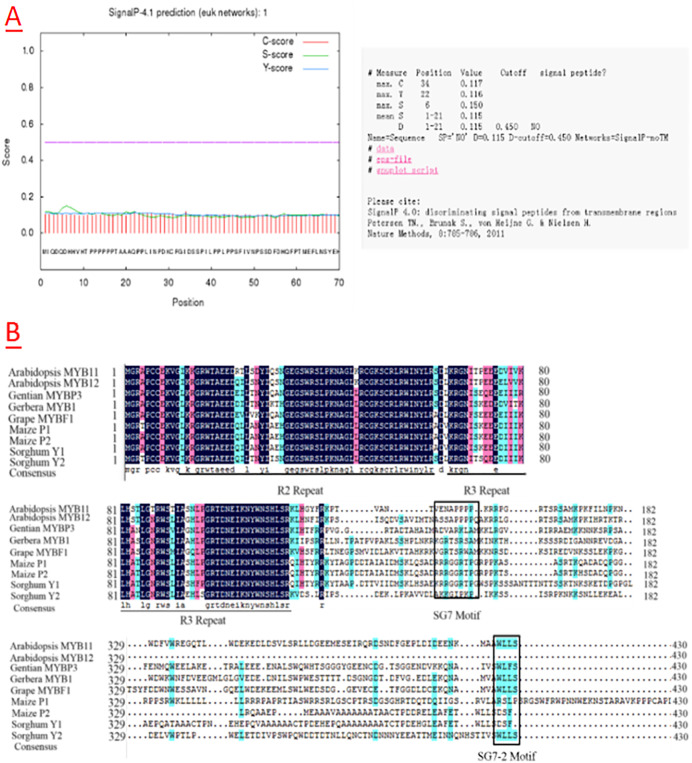
Sequence analysis of *CtMYB1.* A The predicted cleavage site of the signal peptide in *CtMYB1*. B The protein sequence alignment of MYB transcription factors, with R2 Repeat and R3 Repeat being typical domains of the R2R3 MYB transcription factor, and SG7 and SG7−2 being special domains involved in the regulation of flavonoid synthesis.

In this study, a 1223-bp cDNA of the *CtMYB1* gene was cloned from safflower (GenBank KY554784), and sequence analysis revealed a 489-bp open reading frame (ORF) encoding a 162-amino acid protein with a molecular weight of 17878.15 Da and an isoelectric point of 4.82. The hydrophilicity of the CtMYB1 protein was predicted using ProtScale and the gradient average hydrophobicity was found to be-0.307, indicating that the protein is hydrophilic (S1 Fig). The prediction of the signal peptide cleavage site showed C, S, and Y scores of 0.117, 0.150, and 0.116, respectively. As all the scores were close to 0.1, it was deduced that the CtMYB1 protein is non-secretory (Fig 1A).

We selected 9 transcription factors that have been reported to regulate flavonoid biosynthesis, including the maize flavonoid synthesis regulatory genes *P1* and *P2*, and the tomato flavonoid synthesis regulatory gene *SlMYB12*, for analysis. Protein sequence alignment analysis showed that all MYB transcription factors that regulate flavonoid biosynthesis are relatively conserved at the N-terminus, especially in the R2R3 domain, while they differ significantly in structure at the C-terminus. Despite the large structural differences at the C-terminus, two relatively conserved structural domains, SG7 (GRTxRSxMK) and SG7−2 ([W/x][L/x]LS), were still present at the C-terminus (Fig 1B). Studies have reported that the SG7 and SG7−2 domains are characteristic features of MYB-like transcription factors that regulate flavonoids [21,22]. These findings indicate that the cloned *CtMYB1* is likely to be involved in regulating flavonoid biosynthesis in safflower.

### The establishment of a system for flower protoplasts in safflower

**Fig 2:**
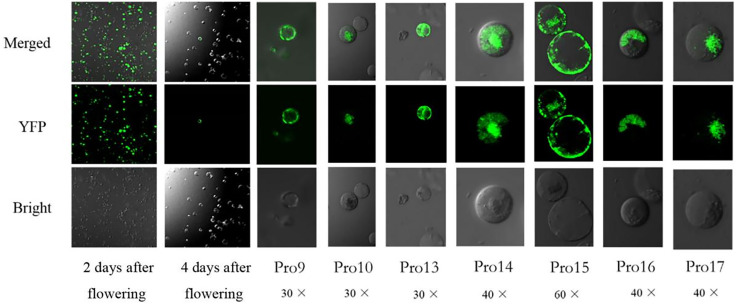
The effect of different times on the transformation of protoplasts. (*pA7-YFP*: Expressed in the whole cell, pro9-pro17: Lysosome, Vacuole, ER, Nucleus, Plasma membrane,Golgi body, Mitochondria).

In this experiment, a system for preparation and transformation of safflower protoplasts was successfully established. The effects of centrifugal force and exosome on the preparation and transformation of protoplasts were explored. We investigated the effect of centrifugal forces of 200 g, 150 g, and 100 g on the state of protoplasts. The results of the experiment are shown in S5 Fig. It can be seen that some of the protoplasts collected at 200 g centrifugal force were broken, the protoplasts collected at 150 g centrifugal force were in better condition, and the protoplasts collected at 100 g had the highest degree of completeness and were more numerous. We explored the viability of protoplasts prepared by sampling 2 days and 4 days after flowering. The prepared protoplasts were transformed with positive plasmids and cultured. Then, the transformation rate was explored by observing the cells under a confocal microscope. The results are shown in Fig 2. It can be seen that the transformation rate of protoplasts prepared by sampling 2 days after flowering was very high and the transformation rate of protoplasts prepared by sampling 4 days after flowering was very low. Therefore, the protoplasts used for transformation of positive plasmids Pro9, Pro10, Pro13, Pro14, Pro15, Pro16 and Pro17 were prepared from safflower at 2 days of flowering. All parts of safflower with ovary removed were cut into 0.5 mm segments for enzymatic digestion. The prepared protoplasts are shown in S5 Fig. It can be seen that the obtained protoplasts contain a lot of pollen grains and other impurities, and the purity of protoplasts is very low.We compared the yield and quality of protoplasts extracted from both the corolla lobes and tubes, and results revealed that a lower number of viable safflower protoplasts was obtained from the lobes rather than the tubes (S5 Fig.). The optimum explant for safflower protoplasts isolation was determined to be young corolla tubes, as they produced a high number of transparent protoplasts after 4 hours of enzyme digestion. Our protoplasts isolation protocol was suitable for subsequent research, yielding approximately 5 x 10^7^ protoplasts cells per corolla tube.

The viability and concentration of protoplasts are important factors in transfection efficiency. In this study, the transfection efficiency of safflower protoplasts was measured by detecting the yellow fluorescent protein (YFP) signal. The stability of the transient expression vectors was verified as the YFP signal could be detected 18 hours after transformation (S6 Fig.). Our results showed that we achieved a high transfection efficiency. The experimental results are consistent with those of Page et al., who found that protoplasts prepared from rice seedlings within 7 days after seed germination had better effects [23], and the 100 g centrifugal force commonly used for the preparation of Arabidopsis and maize nucellus protoplasts [24,25]. The experimental results demonstrated that there were no significant differences in protoplast yield between different batches or replicate experiments.

### Subcellular protein localization of CtMYB1 in safflower flower protoplasts

**Fig 3:**
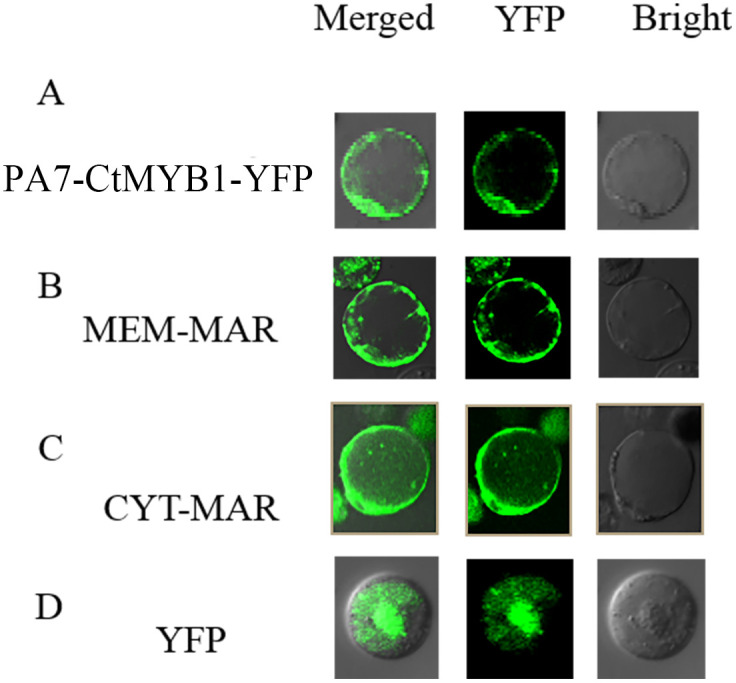
Subcellular localization in safflower flower protoplasts. A CtMYB1-YFP was targeted to the cell nucleus. B A marker for membrane location. C A marker for cytoplasm location. D pA7-YFP was used as the control, and YFP was expressed throughout the entire cell.

It should be noted that there have been no previous reports of using safflower protoplasts to characterizing genes. To evaluate the suitability of the transient expression system of flower protoplasts for protein subcellular localization, we conducted an experiment. Our results showed that the fusion protein *CtMYB1-YFP* was expressed in safflower flower protoplasts and was localized in the nucleus (Fig 3A). To further demonstrate the versatility of this system, we used *YFP* markers for plasma membrane (MEM-MAR) and cytoplasm (CYT-MAR) localization that are reserved in our lab. After 18 hours of transfection, MEM-MAR was expressed in the plasma membrane, and CYT-MAR was expressed in the cytoplasm (Fig 3B and 3C). As a control for the subcellular localization studies, we used *pA7-YFP*, which showed a brilliant green fluorescence throughout the cell (Fig 3D). The results suggest that safflower flower protoplasts can be used for characterizing genes.

### CtMYB1 promotes the expression of flavonoid-related genes in protoplasts

**Fig 4:**
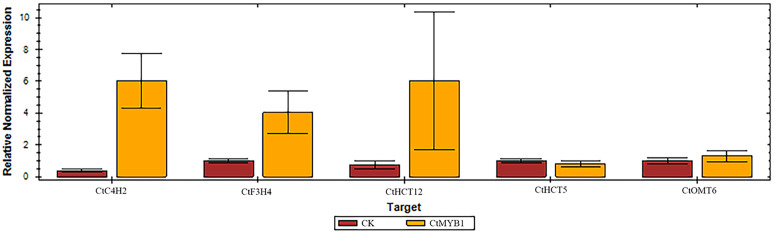
Expression analysis of genes involved in safflower flavonoid biosynthesis after being infiltrated into safflower protoplasts. *CtC4H2*, *CtF3H4*, and *CtHCT12* genes can be upregulated by *CtMYB1*.

The goal of the study was to investigate the potential association between the *CtMYB1* gene and flavonoid biosynthesis in safflower. Based on the transcriptome sequencing results of the previous research [26], five genes related to flavonoid biosynthesis were selected for expression analysis, including *CtC4H2*, *CtF3H4*, *CtHCT12*, *CtHCT5* and *CtOMT6*. After we transforming the *CtMYB1* gene into the safflower flower protoplasts, the RNA was isolated and qRT-PCR was performed to measure the expression of the selected genes. The results showed that the expression of the genes *CtC4H2*, *CtF3H4*, and *CtHCT12* was upregulated by the *CtMYB1*, while the expression of *CtHCT5* and *CtOMT6* remained largely unchanged (Fig 4). *CtC4H2*, *CtF3H4*, and *CtHCT12* are important genes in safflower flavonoid biosynthesis. In our previous report, we found that the increased expression of these genes is associated with the content of flavonoids in safflower under MeJA (Methyl jasmonate) treatment [26]. The results suggest that overexpression of *CtMYB1* in protoplasts upregulates the expression of genes involved in flavonoid biosynthesis.

### Flavonoid synthesis was downregulated in the VIGS system of *CtMYB1*

**Fig 5:**
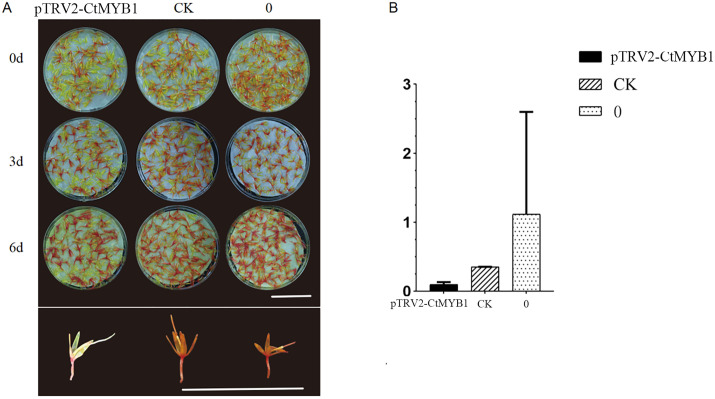
Analysis of components in safflower after silencing *CtMYB1.* A Vertical representation records the color change of Agrobacterium infiltration solution treated safflower after 0d, 3d and 6d. Horizontal representation records the color change of Agrobacterium infiltration solution-treated safflower transformed with p*TRV2-MYB1*, CK and (0) aseptic dip solution-treated safflower. Bar = 5 cm. B Relative content of HYSA in petals after Agrobacterium infiltration. *pTRV2-MYB1*, CK, and 0 were grouped as in A. (pTRV2-MYB1: experimental group, CK: control group, 0: blank group).

After safflower was infiltrated with the Agrobacterium infection solution containing the transformed plasmid, GUS staining proved that the infiltration effect was good(S8 Fig.). Trypan blue staining was used to determine that the safflower was inactivated after 6 days of cultivation(S8 Fig). qRT-PCR was carried out to confirm that the expression of *CtMYB1* was silenced in the experimental group. The color of safflower at the initial flowering stage changed from yellow to red. Compared with the control group (CK) and the blank group (0), the color change of safflower in the experimental group (*pTRV2-CtMYB1*) was less significant. After 6 days of cultivation, the petal color of the experimental group (*pTRV2-CtMYB1*) was significantly lighter than that of the control group (CK, 0) (Fig 5A), which indicated that silencing *CtMYB1* inhibited the color change of safflower. The content of Hydroxysafflor Yellow A (HSYA) was determined, and the results showed that the HSYA content in the experimental group was significantly lower than that in the control group (CK) and the blank group (0) (Fig 5B). The results of the VIGS experiment demonstrated that *CtMYB1* is involved in regulating the synthesis of HSYA, a flavonoid component in safflower.

### CtMYB1 bind to the CAACCA element of flavonoids biosynthesis genes promoters

**Fig 6:**
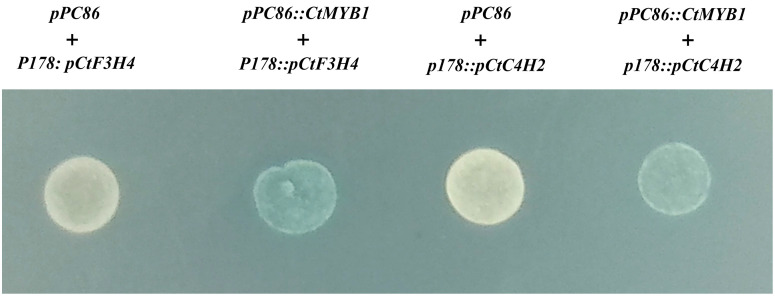
Yeast one-hybrid analysis of CtMYB1 with promoters related to flavonoid biosynthesis genes. Two promoters (*pCtC4H2*, *pCtF3H4*) were analyzed. White color indicates that the promoters themselves do not have self-activation properties. Blue color indicates that there is an interaction between MYB and the promoter.

First, yeast one-hybrid assay was used to analyze whether CtMYB1 can bind to promoters related to flavonoid biosynthesis genes. We selected the promoters (*pCtC4H2*, *pCtF3H4*) of two genes (*CtC4H2*, *CtF3H4*) that are upregulated by CtMYB1 for verification. The promoters were cloned and ligated into the *p178* vector. First, the background expression of the promoters was detected by transforming only the empty *pPC86* vector and the *p178* vector with the ligated promoters. Meanwhile, we transformed the *pPC86* vector containing *CtMYB1* together with the *p178* vector containing the ligated promoters. The results showed that in the double-deficient medium containing X-a-gal, the yeast cells transformed with the CtMYB1-containing *pPC86* and the promoter-containing *p178* vector developed color (Fig 6), indicating that CtMYB1 binds to the promoters *pCtC4H2* and *pCtF3H4*.

**Fig 7:**
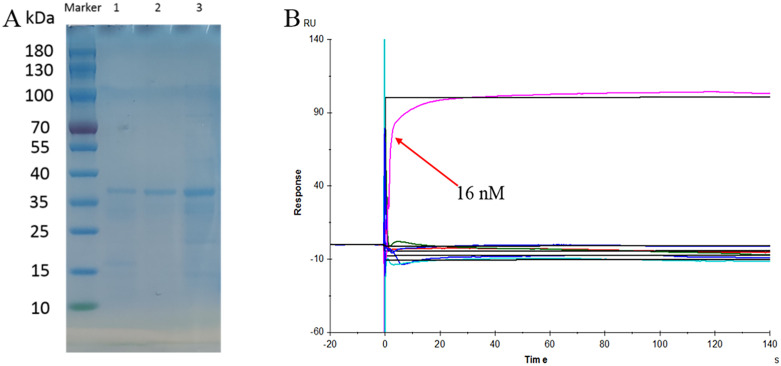
Binding analysis of CtMYB1 with DNA elements. A SDS-PAGE analysis of CtMYB1 fusion protein after purification by Ni-NTA Resin column, with 1-3 representing proteins eluted with imidazole of 160, 80, and 40 mM. B A representative sensorgram of fitted kinetic data from the kinetic analysis of nucleic acids binding to CtMYB1 that has been directly immobilized to Sensor Chip CM5 using amine coupling.

Then, we analyzed the binding of CtMYB1 to specific elements of the promoters. First, the recombinant expression vector *pET32-CtMYB1* was successfully constructed, and the recombinant vector was transformed into BL21 competent cells for prokaryotic expression. SDS-PAGE analysis showed that the CtMYB1 fusion protein had a molecular weight of approximately 35 kDa, suggesting that it may function as a dimer (Fig 7A). The core elements interacting with MYB in 10 flavonoid synthesis gene promoters were predicted. After removing the duplicate elements, a total of 23 elements were finally obtained. The single-stranded promoters containing the core elements were synthesized. After annealing the single-strands into double-strands, the interaction between the CtMYB1 protein and the promoter elements was analyzed using a Biacore T200. First, the coupling results were analyzed. The results showed that 299.3 RU of ligand was coupled. The multi-cycle measurement results showed that the CtMYB1 protein specifically binds to the MYB element “CAACCA” on the promoter of the flavonoid synthesis gene, as shown in Fig 6. At this time, the concentration of the analyte was 16 nM, and the dissociation constant KD was 1.180 × 10^−9^ M. The results showed that the core element of MYB, CAACCA, could directly bind to the CtMYB1 protein immobilized on the sensor chip surface. The binding concentration was 16 nM (Fig 7B).

## Conclusion

The use of protoplast transient expression systems has been crucial in plant research for studying gene functions, protein subcellular localization, and interaction assays. For instance, the mesophyll protoplasts transient expression system of Arabidopsis is a highly efficient and important tool for gene function research [27,28]. Similarly, the versatile and physiological protoplasts system of rice has been demonstrated to be useful for research on light/chloroplast-related processes [29,30]. However, heterologous systems may result in inaccurate findings due to the presence of a foreign genetic background. Thus, homologous transient expression systems are much more accurate for gene function research. The successful isolation of high-yield and viable protoplasts is critical for the protoplasts transient expression system. Protoplasts freshly isolated from leaves have been shown to be a significant tool for gene function studies [31,32], and a transient expression system using root protoplasts has also been established [33]. In contrast, the isolation of protoplasts from safflower flowers is challenging due to the structure of its corolla. Therefore, the proper selection of the explant is crucial for obtaining high-quality, viable protoplasts for subsequent experiments.

This study established efficient methods for isolating flower protoplasts, using VIGS, and performing transient expression. The results showed that the *CtMYB1* gene is capable of regulating the expression of genes involved in flavonoid biosynthesis in safflower. Inhibiting the expression of *CtMYB1* inhibits the color change of safflower flowers while significantly reducing the content of HSYA in the flowers. The effect of the transcription factor *CtMYB1* on the expression of flavonoid synthesis genes was analyzed by qRT-PCR. It was found that *CtMYB1* upregulated the expression of the genes *CtC4H2*, *CtF3H4*, and *CtHCT12*. In the flavonoid biosynthesis pathway of safflower, multiple HCT genes are involved. In the experiment, the *CtHCT5* and *CtHCT12* genes showed different expression trends and intensities. Among them, *CtHCT12* may play a more significant role [34]. It is speculated that in this experiment, the *CtHCT5* and *CtHCT12* genes function in different branches of the flavonoid biosynthesis pathway of safflower. Combining with the flavonoid biosynthesis and metabolism diagram of safflower (S9 Fig), it is speculated that after overexpressing *CtMYB1*, the expression of the *CtC4H2* gene increases, promoting the accumulation of p-coumaroyl-CoA, leading to the accumulation of naringenin chalcone, naringenin, etc.; the expression of the *CtF3H4* gene increases, promoting the accumulation of dihydrokaempferol, kaempferol, etc.

In addition, the sequences of 10 promoters were analyzed, and finally 23 MYB elements were predicted. After synthesizing the promoter fragments, the interaction between the *CtMYB1* protein and the promoters was analyzed using a Biacore T200 interaction analyzer. It was found that the *CtMYB1* protein interacts with the “CAACCA” element. Based on the analysis combined with the qRT-PCR results, it was concluded that the *CtMYB1* protein binds to the MYB element “CAACCA” in the promoters (both the *CtC4H2* and *CtC3H1* promoters contain this element), regulating the expression of the *CtHCT12*, *CtC4H2*, and *CtF3H4* genes, and thus affecting the biosynthesis and metabolism of flavonoids. This study provides valuable insights into the molecular mechanisms of flavonoid biosynthesis in safflower, and the information gathered here will be valuable for future research in safflower.

## Supporting information

S1 Fig Bioinformatics analysis of CtMYB1. A The prediction result of CtMYB1 Hydrophobic map. B The prediction result of transmembrane helical structure of CtMYB1.
(TIF)

S2 Fig Structural Analysis of CtMYB1. A The prediction result of CtMYB1 secondary structure. B The prediction result of CtMYB1 tertiary structure.
(TIF)

S3 Fig Neighbor-Joining phylogenetic tree. (TIF)

S4 Fig Overview of the separation and pretreatment of safflower flowers. A The separation of the corolla. B The gathering of the corolla in 0.6 M mannitol for osmotic treatment. C The enzymatic hydrolysis of the protoplasts. D The collection of the protoplasts.
(TIF)

S5 Fig Comparison of the yield and quality of protoplasts extracted from different parts under different centrifugal forces. A Protoplasts isolated from the corolla lobe. B Protoplasts isolated from the corolla tube.C The effect of different centrifugal force on protoplasts.
(TIF)

S6 Fig Protoplasts transfected with pA7-YFP. The YFP signal is detected 18 hours after transformation.
(TIF)

S7 Fig The result of CtMYB1 subcellular localization. (TIF)

S8 Fig Establishment of Virus - Induced Gene Silencing (VIGS) System in Safflower. A Agrobacterium-mediated GUS staining of safflower. B Results of trypan blue staining.
(TIF)

S9 Fig Pathway of safflower flavonoids synthesis (incomplete). (TIF)

S1 Table Primer sequence. (PDF)

S2 Table Primers for CtMYB1 Vector Cloning. (PDF)

S3 Table The primers for qRT-PCR. (PDF)

S4 Table Promoter sequences of 10 genes related to flavonoid synthesis. (PDF)

S5 Table MYB element and synthetic sequence. (PDF)
