# Supplementary figures and images for: CtMYB1 regulates flavonoid biosynthesis in safflower flower by binding the CAACCA elements
Source: PLoS One. 2025 Dec 10;20(12):e0337921. doi: 10.1371/journal.pone.0337921 (PMC12694881; doi:10.1371/journal.pone.0337921)

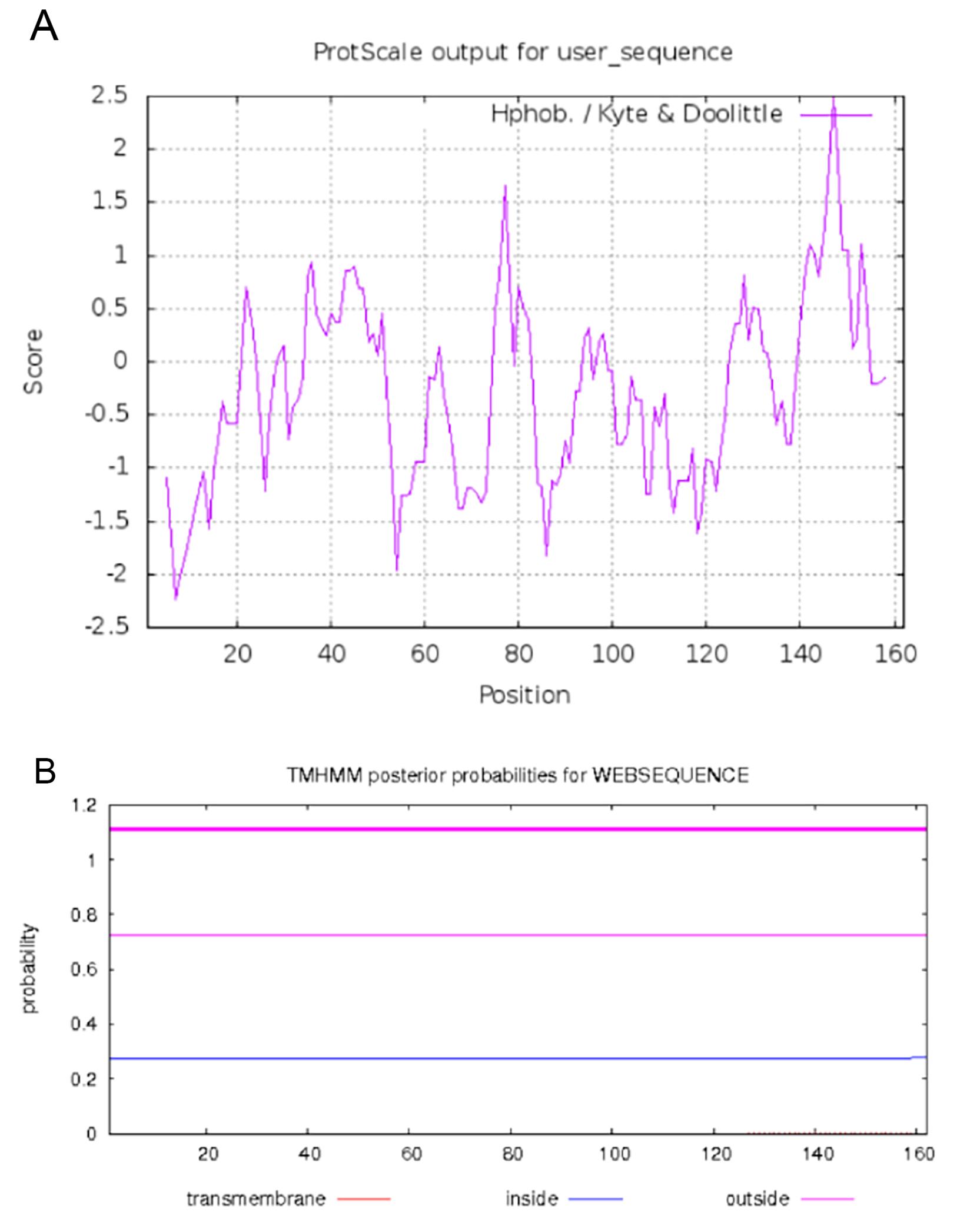

Supplement: S1 Fig — A The prediction result of CtMYB1 Hydrophobic map. B The prediction result of transmembrane helical structure of CtMYB1. (TIF) [file pone.0337921.s001.tif]

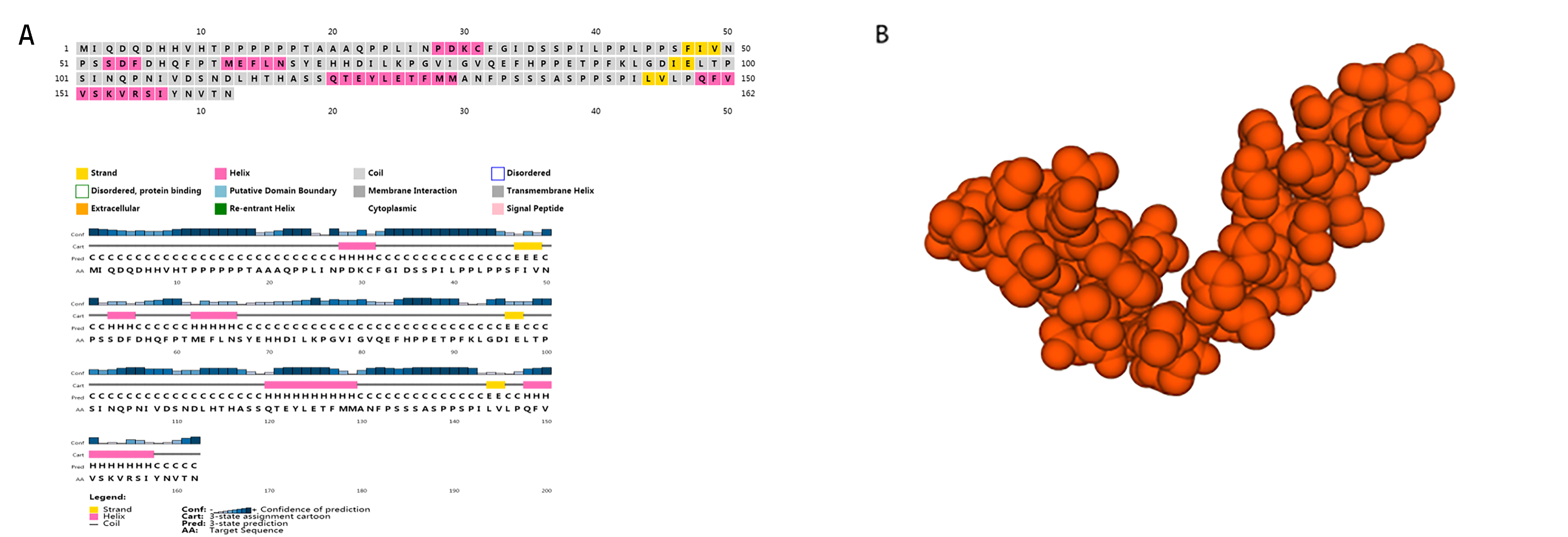

Supplement: S2 Fig — A The prediction result of CtMYB1 secondary structure. B The prediction result of CtMYB1 tertiary structure. (TIF) [file pone.0337921.s002.tif]

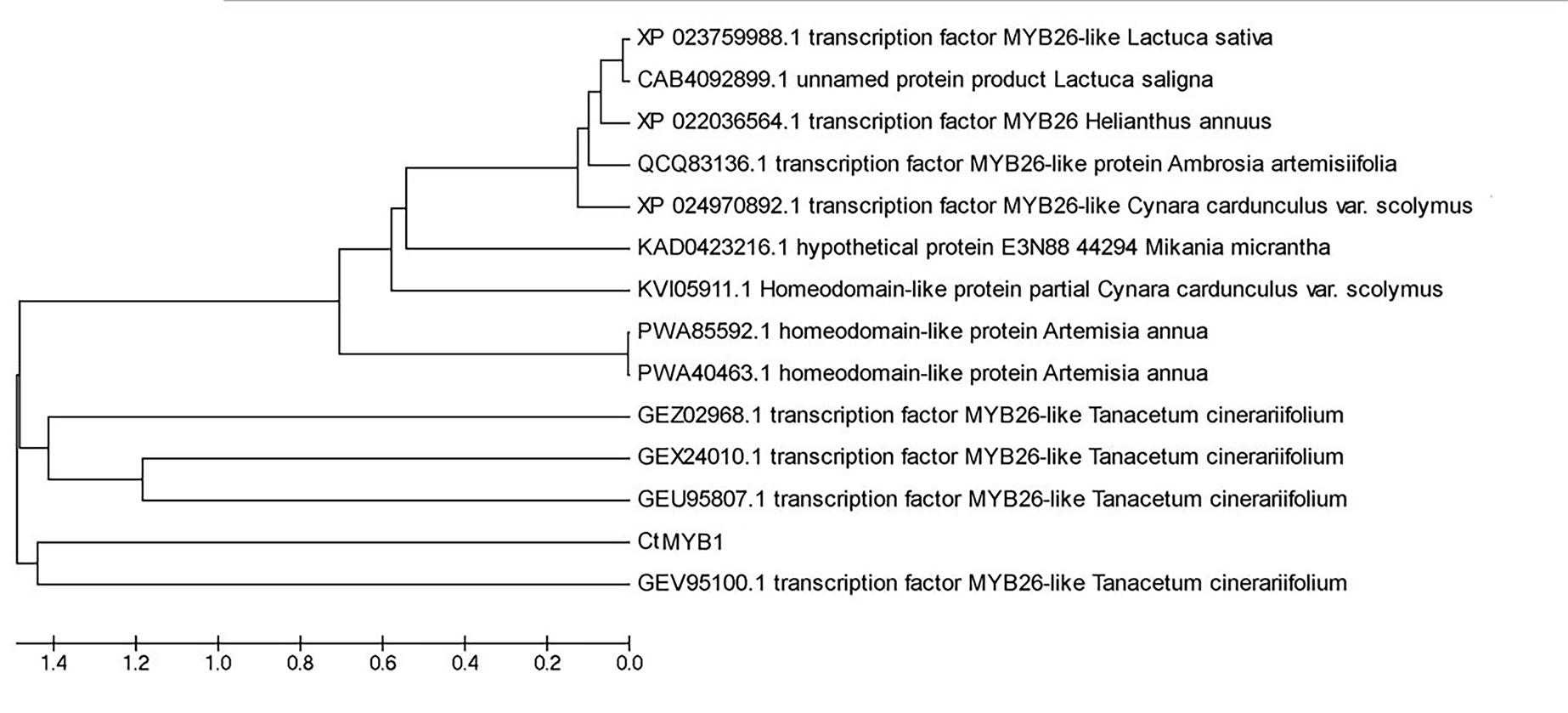

Supplement: S3 Fig — (TIF) [file pone.0337921.s003.tif]

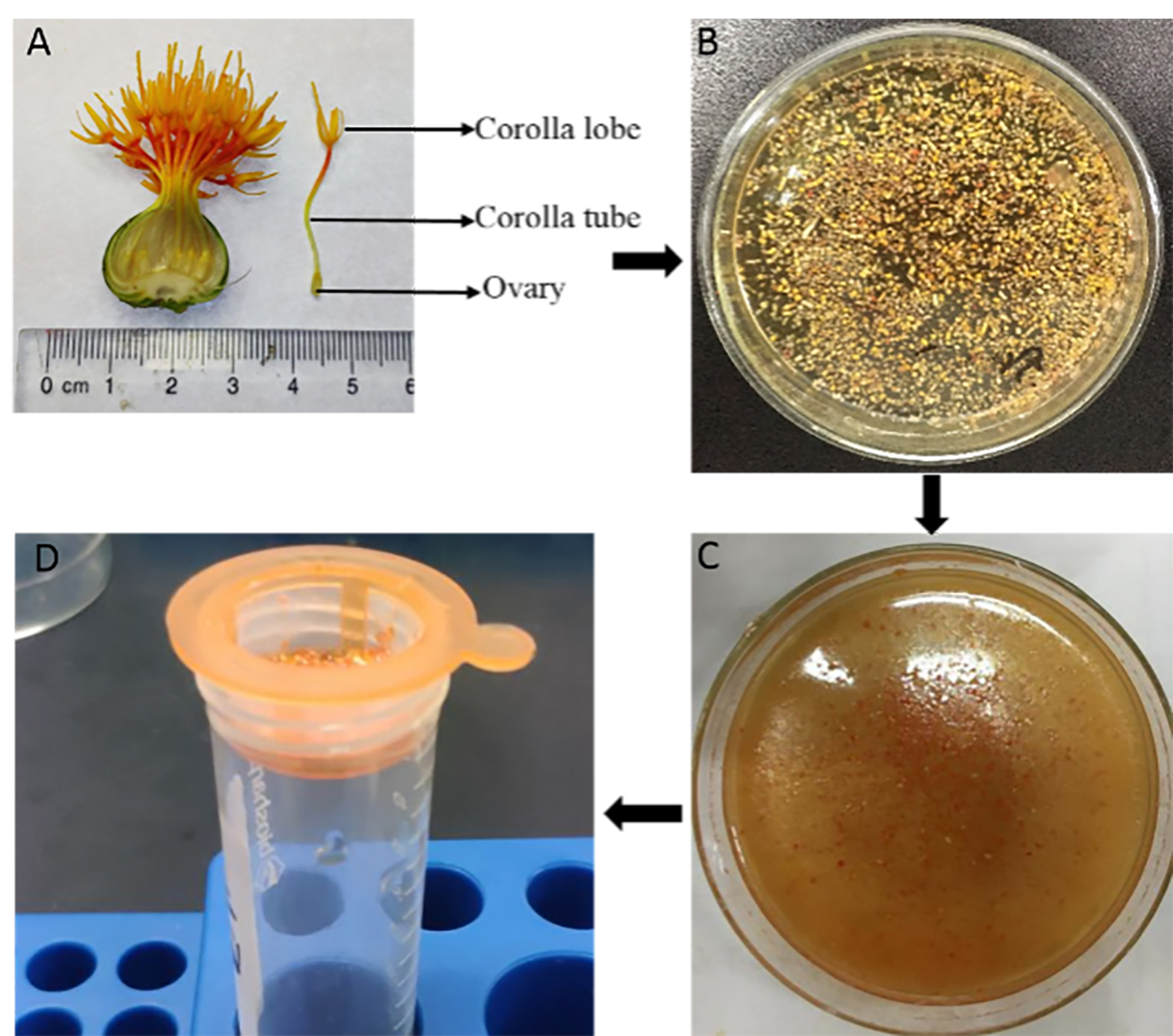

Supplement: S4 Fig — A The separation of the corolla. B The gathering of the corolla in 0.6 M mannitol for osmotic treatment. C The enzymatic hydrolysis of the protoplasts. D The collection of the protoplasts. (TIF) [file pone.0337921.s004.tif]

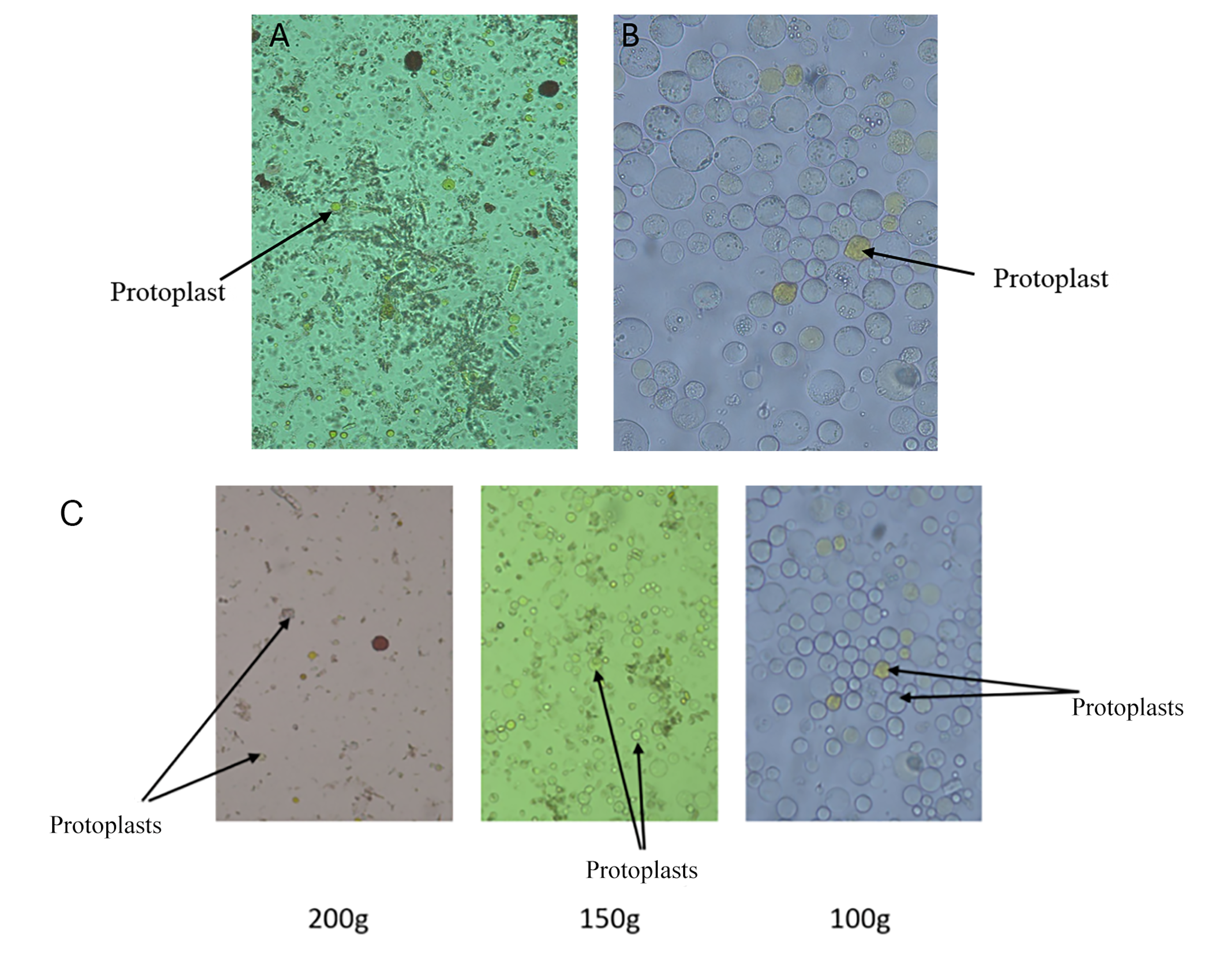

Supplement: S5 Fig — A Protoplasts isolated from the corolla lobe. B Protoplasts isolated from the corolla tube.C The effect of different centrifugal force on protoplasts. (TIF) [file pone.0337921.s005.tif]

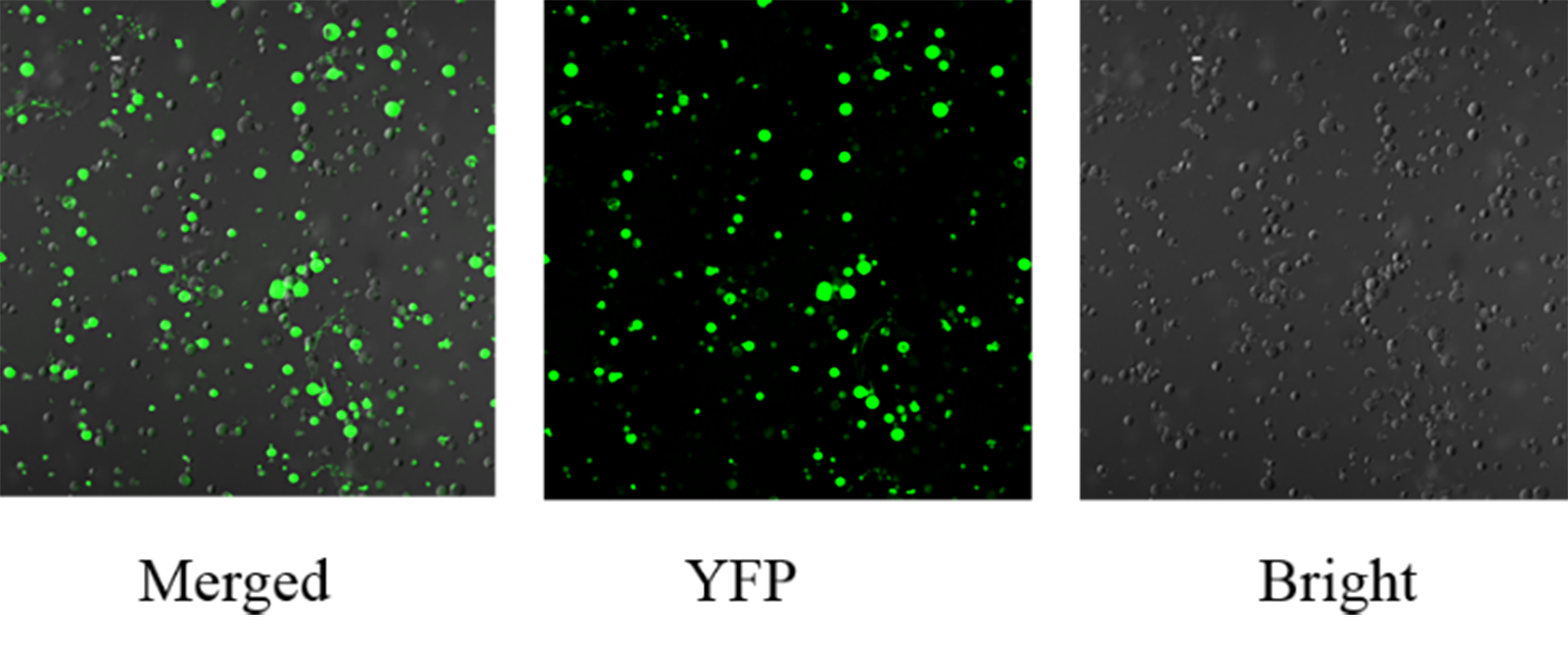

Supplement: S6 Fig — The YFP signal is detected 18 hours after transformation. (TIF) [file pone.0337921.s006.tif]

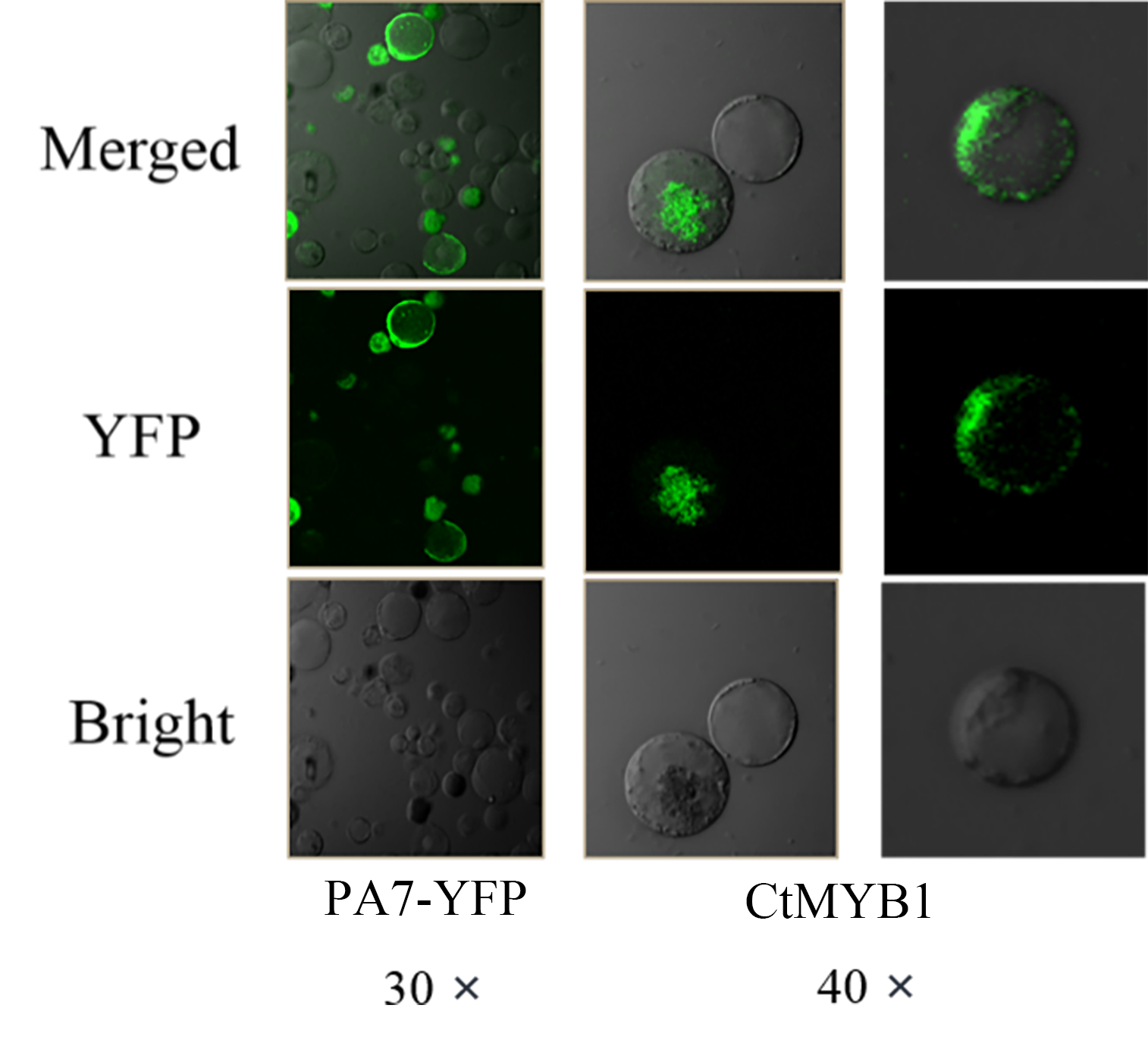

Supplement: S7 Fig — (TIF) [file pone.0337921.s007.tif]

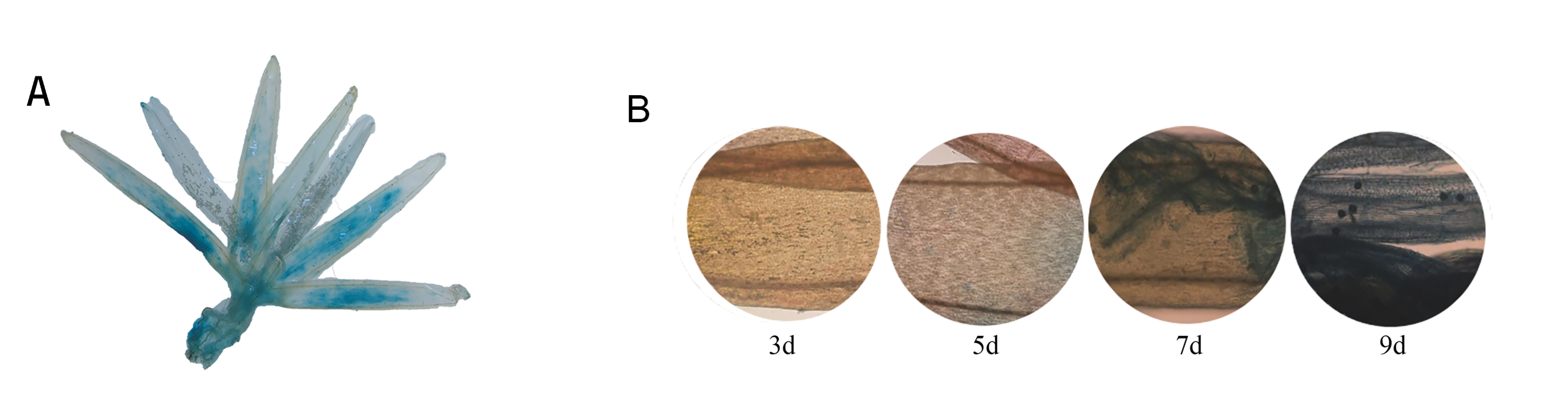

Supplement: S8 Fig — A Agrobacterium-mediated GUS staining of safflower. B Results of trypan blue staining. (TIF) [file pone.0337921.s008.tif]

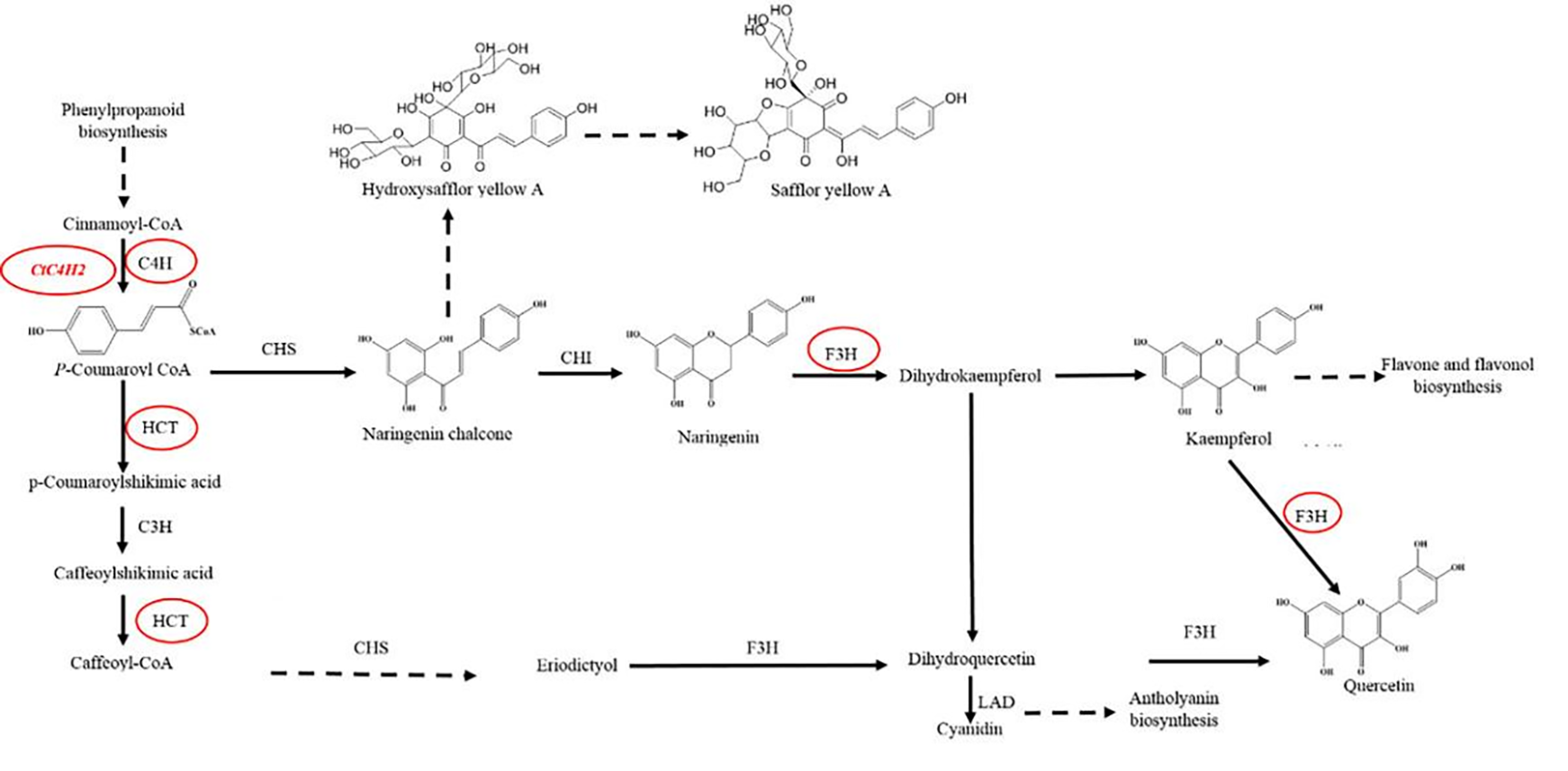

Supplement: S9 Fig — (TIF) [file pone.0337921.s009.tif]
