# Supplementary material for: CtMYB1 regulates flavonoid biosynthesis in safflower flower by binding the CAACCA elements
Source: PLoS One. 2025 Dec 10;20(12):e0337921. doi: 10.1371/journal.pone.0337921 (PMC12694881; doi:10.1371/journal.pone.0337921)
Supplement: S1 Table — (PDF) [file pone.0337921.s010.pdf]

S1 Table . Primer sequence

Names	Sequences of the Primers
***CtMYB1* sequence**	F：ACTCACCTTATTAGTTAGAGA<br>R：ACATGCAAGGAAACAGTAATT
***CtMYB1* coding sequence region(CDS)**	F：ACCCCACATGATCCAAGATCAAG<br>R：TAACATTGATTAATTAGTCACAT
