# Supplementary material for: CtMYB1 regulates flavonoid biosynthesis in safflower flower by binding the CAACCA elements
Source: PLoS One. 2025 Dec 10;20(12):e0337921. doi: 10.1371/journal.pone.0337921 (PMC12694881; doi:10.1371/journal.pone.0337921)
Supplement: S2 Table — (PDF) [file pone.0337921.s011.pdf]

S2 Table . Primers for *CtMYB1* Vector Cloning.

Plasmid vector	Sequences of the Primers
pET-32a	F：GCCATGGCTGATATCGGATCCATGATCCAAGATCAAGATC
(+)-CtMYB1	R：ACGGAGCTCGAATTCGGATCCTTAATTAGTCACATTATAT
pA7-CtMYB1-YFP	F：TTCCTGCAGCCCGGGGGATCCATGATCCAAGATCAAGATCA
	R：ACTAGTATGGTGAGCGGATCCATTAGTCACATTATATATAC
pTRV2-MYB1	F：GTGAGTAAGGTTACCGAATTCATGATCCAAGATCAAGATCA
	R：TGGAGGCCTTCTAGAGAGAATTCATTAGTCACATTATATATATAC
