# Supplementary material for: CtMYB1 regulates flavonoid biosynthesis in safflower flower by binding the CAACCA elements
Source: PLoS One. 2025 Dec 10;20(12):e0337921. doi: 10.1371/journal.pone.0337921 (PMC12694881; doi:10.1371/journal.pone.0337921)
Supplement: S3 Table — (PDF) [file pone.0337921.s012.pdf]

S3 Table . The primers for qRT-PCR.

Gene name	PCR primer
Ct25s	F：GGAGGTTGAGGGAAAAGGAG
	R：GTGACCTCGTCACCCGTAGT
CtHCT5	F：AACCTTCCAGCCCTACGC
	R：AATCCGACTCCGCCTCAA
CtHCT12	F：ATGGCATCTTTACAAATAACCG
	R：CAACTCCTAGCGACACCC
CtC4H2	F：AAATCCAAGCGAAACTGA
	R：GCAAACTAAACTGCCCAC
CtF3H4	F：ATCTCAGAAGCCAGCAAA
	R：GCCAAACCCTATGAAACA
CtOMT6	F：TTCTTTCTACCGCCCTTGC
	R：TCGTAGCCGATGACTCCC
CtMYB1	F：ATGATCCAAGATCAAGATC
	R：TTAATTAGTCACATTATAT
