# Supplementary material for: CtMYB1 regulates flavonoid biosynthesis in safflower flower by binding the CAACCA elements
Source: PLoS One. 2025 Dec 10;20(12):e0337921. doi: 10.1371/journal.pone.0337921 (PMC12694881; doi:10.1371/journal.pone.0337921)
Supplement: S4 Table — (PDF) [file pone.0337921.s013.pdf]

S4 Table.Promoter sequences of 10 genes related to flavonoid synthesis

pCtCHI3

TGTTAATTGGAGGATAGTTGGAGTAGCACTTTAGGAGCTTTTTCTAATTATCCCTTTATTAATGTTAATCATTATATCCTAAGACTAAGA
TATATCCTTTTTAATCATTTTACATATACATAAGCTAGTTTTTATCAAACATAATTTATTCATAAGCTACCTTATCAGCTAGCAGCTACCT
TATCAGCTAGCAGCTACCTTATCAGCTAGCAGCTACCTTATCAGCTAGCAGCTACCTTATCAGCTAGCAGCTACCTTATCAGCTAGCA
GCTACCTTATCAGCTAGCAGCTACCTTATCAGCTAGCAGCTACCTTATCAGCTAGCAGCTACCTTATCAGCTAGCAGCTACCTTATCA
GCTAGCAGCTACCTTATCAGCTAGCAGCTACCTTATCAGCTAGCAGCTACCTTATCAGCTAGCAGCTACCTTATCAGCTAGCAGCTAC
CTTATCAGCTAGCAGCTACCTTATCAGCTAGCAGCTACCTTATCAGCTAGCAGCTACCTTATCAGCTAGCAGCTACCTTATCAGCTAG
CAGCTAGCTTATCAGCTAGCAGCTACCTTATCAGCTAGCAGCTAGCTTATCAGCTAGCAACTATTCGTTTAAAAGTTTTCAAAATGGT
GTAGCCATTGGCAGTTGGAAATGCCTTTGTTCTTAATTCCACTGTTCTTTGTTGGCTTTTGACCATCCACAATGGTCCGGTAAAGCCA
TCATCGGCATGCTGCTGTTGCCGATCGGTAAGAGTGGCAACACCGTCGAGGTTGATTGACAAGCACCAAAATCGATACCCCCATGC
CGACTCCTTGGATGATCCACCCCACCCTCTATCTCCTTTCTCTCTATCTCTCTCTTCTTTCTCTCTCTTCTCTTTTTTTTTTCCTTTTTTA
TTAATTATTTAGATTGGTGTATGTTTGGTTGAATTGGTAAGATAATGGAAGACTGATGTCATTGGCAAGAGCCGTCATCATTGTGGAT
GATCTTAGTTTCAAAGACCATTGCCAATAGGAATGACAATGGGGCGGGTTCGGGGCGGGGCAGGCTATCCCCGTCCCTGAACCCGA
AATCGAAAATCTGCCCTGTCCCCCGTATATGTCCTGTACTAATCCACTTCTCTTTGTGTACACCAGGATTTCTTAACTCTAATTTTTAA
TATGATATCGGGGCGGGGCGGGGCACGGTCACCCCAGTCACTGCCCCGAACCCCGAAAAATTCTCCGTTAACACCCCGATCCCCGC
CCTGTAACCCGATAATGCGTGCCCCGCCCTGCCCCGTTAGGATCGGGTTTCGGGTTTCCCCGTCGGGTTCAGATTTTTTTGCCATAAT
TGCCAACAAATCATTTTTTCTCTTTTTGTGTAACTTGAATAAAATTTTGTACATTTATCATCCCAACCGTCCATATGGTCCGACCCGCT
TCTTTTCTTCCACACGATCCATCTACCACTTCCATTTCCTCTATAAATGCCTACCCACCACCTCCCTACCGTTAACTGTCACTCGCTCA
CCACAATTCAATTCTTCTTCCTGTTTCTCCGACTCAACTACA

pCtC3H6

ACTATATACAGATTACAAGATAAACAATAAGGAATAAATCTTCAAAATCTTTAATATCTGCTCCAAGATCTTCGTATTTCTTTCTT
ATCGCAATCTTCAAAAGCTTTCCATATTGCAAACCAACTAGCAGTGTTAAACACTAAAGCAGTGTTAACTTTGTACTAGCAGTGG
TAATCTGATCTGGCAGTGGTAAGATGAATAGCAGCGATAACCTGCAGTGTTAACAACACTAGCAGTGGTAATCCTTTACCAGCAG
TGGTAACTTCCTGCAGCAGTATTAATTTCTTCAAGCAGTGGTAAATGTTCAGCAGTGGTAACTCTTCAATCCCAACATTTTACATA
TTCATAAGCTAGTTTTTATCAAATATAATTTGTTCATAAGCTATCAGTTAGTTTGTCAAATATAGTATCAAAGGGTGTTAGCTCAG
TTGGATAGGTAACATCCCTGAGGGGTGTGGGGTGAAAGCCACACTACCAGGGTTCGAATCCCACGGGTGACATTTCTGAGATAT
CCAGACTTTGGATCTAGTCCTTTAGGCCGAGTTCGGGGCCGGTATGGGAGGATCCAGTCTATTGGATTCACTTCCCTGCCGGTCA
GGTTCAGCCATAGATTGGACCGGGTCACAGCGGATTAGTGGGATTGCCCAATTAAAGGTCCGATACCGCTGGAGTATGTATATGT
CCACTTGCGGTTTAAAAAAAAAGTTTGTCAAATATGGCCTTAATAATAAAATGAAGCCCACATTGATTAAAAGCCATTTATTAGA
ATTCAACACACATTGAGTTTGGTAGTGTTTTATGGGCCCTTTTCAAAATCATTTTCAATATATTATTAACCTGCAATTGTTTTTTA
AGATAACTTATTAACCTACAAGTATTTAACATTATTATATTATTTTGGACAGACATATCATATTATTATATCAAAATCTTTTTTGA
AATTACGAGGGTTAGCAGCTGCTAACTCTTGTTACCATCTTCCTCTGCCCCTGTCTTACAGTAATTACCGTTACGCTACAAGCCTT
ATGGTTAACAACTTTTAGGACAACTATTTATTATAATATATCATTTTTAGGCACCTATTAAAGCATGTTTAGTCCAGTGGTAAGGT
AACATCCTGGGGAGATCAGTGGATGGTATATCTAACTAGCCGGGTTCGAAGATGAGCATATAACAATATCTAGATCATAGGCAT
ATGCTAGCAGTACACTTAATTGGATCACATGGATTAGTTGGATACCCTTAGATTCCAGATACCTCGAAAAATGTGTGTTAGCTTA
TAGTTTAAAGTAACATTTTTTAATGATTATTTCTTTGAAGTAATGTGGATATATGTCGGCAAACCATTGAAGGTGTGTGATAATCG
CACCTAGGGTGTTAACAAGTCGAGCCGAGTCGAGTATTGGGTTAATCGAGCCGAGCTTAAAAAATAATACTGGGCACGAGCTCG
ACTCGAGCTCGATTAACTTTTTCAAAGTACGGCTCGAGCTCGAGCTCGAATATTTCTTGAGTTATCAGGCTCGAGCTTGAAGCTCG
TTTATAGGTTCGAACTCGTGATCGATTAACTCGTTTATCAACTTAAGCTCGATAATTTTTATATTTTTATACTATTTCTTAATAAAT
ATATGAAAATATTTAGCTCAAACAAAGGTATGCTCGAATGGCTCGGTTTGGCTCGAAAAATTTAATCGAGTTTTCGAACGAGTCG
AGTCAAACATTGGTGTTATAAAGCTCGGCTCGTTAGCTTTTCAAAAAAAAAAGCTCGGCTCGTTAAGTTAAACGAGCTCGATTTT
ATGTTCGAGATCGACTCGAGCTCGAGTATTATTAAGCTCGAGCTCGAAATAAACGAGCTCAAGCTCGAATAGCTCGACTCGAAC
ATTGGTGTTATAAAGCTCGGCTCGTTAAGTTAAATTATCGGTGGGATTCTCACCGATAATTTTGCCAACCATTCAAACATCGTTCC
TATATAACCCTCCCACCCTAAACCCCCCAAGTAGTGGAACTCAACCCCCTCATTTGTGAA

pCtF3H3

TGGGGATCAATTTCGTTGGTTTATATAAACCTTAGGGACCAAATTTATAGTTTACTCATAATAAATGTATTATGGTA
TTGGCATTTAATAAACGGCGGCGATCTTTACACCATATAATTTAATATGAAAAATAATGGTCATGAAAAAAGATTT
TCATACATATATATATATATATAGAGAGAGAAAGGTTCAAATGAGAAGTGCTAAAAAAATGCGAAGTAGAGAAGT
GATCTCCACCACTCATACGGTCAATAAATGAGGCTTTGATCGGTCCACCACTCATACGGTGGACGTGATCACGG
AGGATCTGTCTCTTATACACATCTAGATGTGTATAAGAGACAGCCGTATGAGTGGTGGAGATCACTTCTCTACTT
CGCATTTTTTTAGCACTTCTCATTTGAACCTAATACTATATATATACCTGTCTCTTATACACATCTAGATGTGTATAA
GAGACAGCCGTATGAGTGGTGGAGATCACTTCTCTACTTCGCATTTTTTTAGCACTTCTCATTTGAACCTAATAC
TATATATATACATACATATATATATATATATTTATACCGAAAAACTGAGTAACCACCAAATGCATTTGTGCATACGGG
TAATTTGTAATATGCACACATGCATAAGGAACACCTTCGTAGCCATTCATTTGGACTGTATGTATTTGAATATAAA
AGTAATTTTTAAAAAATTGTAATATGCAGGTGTGCAAAAGAAACACCTTTGTAGTCCTTTATTTGGACCGTATGC
ACTTGTACATAGAAATAATTTGTAATATGTAAATGTCCATAGTCCTTAAGTGCATAATTATAAGTACATAGATACAA
TCTACATAAAAGGCTATAAAGGTATTTCTTAATTTTTTTTCTTATAGAGAGAGGAAGAGAGTGTGTGTATAGTTC
ACACAAAACCGAAAAAGAATAAAAAAAATGAGAAATACCCTAAATAACCTTTCATTTATACAATTACATATAATT
AAATAATTGAACATGGTTCCGTTCGGGTTAGCAGGTTGGCGGGTCAAAAACCTCAACCCATGACATGACCCTTT
TAGCTAATCGTGTCGAGATCTTCGACCCTTACCCGTGATGGGTCGTGTCACGGGTTGGCGAGTCAGAAACGGGT
TGCTGACGGCGACGACGACTGGAAGAATGGAAGGCATGAGCGAGAGAGACGATTGGCATTTGGAACGGAAAG
TGAGATTGAATGTCGCCTGCTTATTGAATATTTTAGGTTACTTAGGGTTATTCACTATATTAAAAATATATAAAAAA
TAAATAAGTAAAAGTTAAACGAGTTCACGGGTGAACCCGTCAAACCCGGCGGGTCAACCCGAACACGACTCG

CGAAATTGACGAGTTGGTGGGTTCGCGGGTCAAAAACCTCAACTCAAACCCGTTTATCGTCGTGTCGTGGCGT
ATCCGGATGGCGGGTCGTGCCGGAAATTGCCACCCCTAATATCATACATATAAATGATTACCAACGCGTTCCTTA
GTTTTCCTCTTTTTCTACATTTCCCTACCTAACCCCTCTCTCCCTATATATATGCATATGATTGTGTATCTACACTTA
AGAAAGATAGCAAAACCACAACCCAAACATCAAAATGGAGGTACAGAGAGTTCAAGAAATAGCCTCACTTTC
AAGCCTAAATGGCACAATCCCAAAAGAGTACATAAGAGCAGTGACTGAACAACCAGCCATCACCACCATCCAT
GGGGTGTTGTTGGAAGTTCCGGTGATCGATCTCAGCCTCCCGGATGCCGAAAAGCTGGTAGCCTCCATCTCAG
AAGCCAGCAAAGAATGGGGCATCTTTCAAGTGGTAAACCATGGGATACCAAGTGAACTCATAAGCAGGTTACA
GAAAGTTGGAAGAGAGTTCTTTGAGTTGCCACTAGAAGAGAAGGAAACCATAGCCAAGCCTGCAAATATGAA
AGATGGTATTGAAGGCTATGGAACAAAGCTTCAGAAGGAGGTTGAAGGGAAGAAAGGGTGGGTGGACCACTT
GTTTCATAGGGTTTGGCCACCTTCTGTCATTAACTACAACTTTTGGCCCAAGAATCCTCCTACTTACAGAGAGAT
AAATGAGGAATATACACAAAAGTTGATAGAGGTGTCAGACAAATTGTTTGGATTTCTATCAAAAGGACTTGGGC
TTGAAGAGAATAAA

CtCHI2

AATCGCATCTCCACCCCCAGTGAATTTATTTAGTGTTGATATTCAATTAGGTTTTTTGATCCAAAGATGAAGATAT
ATTCTTACCTTTCTTACCATTACTTTTCTTCTCATCAGAATCCAACCTAGGATTTAATACATATGGTTGGGTTCGGT
GATAAAGGATAAGAAGGGGTCTTCATCAGCGAATCAATCCTAATTTGAAGCAAACAGATCATTCACACTATTTA
ACGATAACTTCCTCAAAAAAATTAACCTCAATGGGGCAGCCTACTGGTTAAGACTTGGTGTTGGGAAAAACCC
ACCCAAGTTTGAATCCGGGGGTGAGCAACCAGGAACCACTGAGTATTAATTCTAGGGGAGCATGCTTTAGCTCT
TGGTAAAAATTACCAAGGATGTAGTCTGGACAAACCAAGATCAACCTTATAAAAAAAACTTCCTCAAAAAATA
AATGAATTAGTTGGATCAAATATGTTACATAAACCAAAAAGAACATAAAATTGTGGGTCCATGGACACAGTTGC
CAAGTCATACTTCCCTATCATCTTTCCTCTCTAAGTTTCAAATGACATTGCCAATTAACTTTTTTCTAATGACTTTT
TTGTTTAACTTTAATTTAATTATTCATATAGTACACTAAAACCAAATATCAATCAACCTCTTTTTTGTACAGATATG
ACGGTTTACTAACCTCTTACTGGATAAGTGTTTATCGATAAAAACATTACCTCACTTACCTTAATGTATAATGTTC
TCGGAGAAAGGTTCAAATGAGAAACATAACTTTTTAAGAAAACGTGAGGAGCAATCTCAGCCACTCATCCGGT
CAACAAAGGCGGTTTTGACCGTCGTCCCCCCTTTAATAGTCTCTACCCTCCCATGAATCGCGTCAATCCTTCGTG
ATCACGTCCACCGTAACCCAAGATGTGTGACTGAGATTGCTCCTCGTTTTTTCTTAAATTGACATGCTTCTCGTA
TGAACCTCCCTCAATGTTCTCGACCATCAAAATTGATTGTTGATAATCATATTCAACCCGACAAACACTTACCGG
ATAAGAGTCTTTTCCCTCACTTTCTTGGTAAGAAGTTCGATCATTGTTATGCCCAGCAGTTTCCATACTCAATAGA
TAAGCACCGTAAAATAAATGAAACAGGAAAACACTTACCTGAATAAGTGTTTAGTAAAGGACGTCTTCTTATAT
AAGTTAATAATATTATTGAATAAGCTTTGAGCGCCTACTTGGTCTGACCTTTGCATCATTTCCCATTTCCTCTATAA
CTATAAAAATAGCTACCAGCCTTCCTTCTGGTAGTTAAATTGGTCAACACCCCATTTACTCACCGGAAGTGCAAT
TACCATGGCTCCGCCGCCGTCCACCACCACCAGTCTTCAGGTCGAATCCGTCGTCTTTCCGCCATCCGTCAAGG
CCCCGGGCACAGCCACCACTTTGTTCCTTGCAGGCGCAGGTACTCAAATCTCAAAATCCGATCTTCAACTGACA
TTTAAGTTTCGATTAGGTGTACAGTTTTTTTTTTGTTTGAACGCTGGATAATTCACGCACATGTCCCGACACGAA
GTCGACCAGGTATCCGGGATCCATTCGGGCACCCGACTAATCCTCGGCAACCATGGACCAACCCGAAAGCAGG
GTATCGCGACATGAATCAAACCTGGATAGATAGGGATGCCATCCAGTGTCCCTCCCAAGGATGTTACCAAACCA
CTAGGCTACTAATGCAGGTGTAGGGTTTTTATTGATTTGAATCCTGTTTCTATAGGTGTGAGAGGTATGGAAATC
GAAGGCAAGTTCGTCAAGTTAACGGGGATTGGCCTGTATTTAGAGGATAAAGCCATTCCGTCACTCGCCGTTAA
GTGGAAGGGCAAAACCGCGGCTGAGTTGATGGACTCCGTCCACTTCTACAGCGACATCATTAATGGTAATCGTC
AACTTATTTTCGGAATCAAAAACTTATACAGATCGATCCCAAGGAATCGATGTCCTctTTTAGATGCAATTATTTAG
GCGTTTTTTTTTGCATCGAACCAACGAATGACTACCACGAGCACATACACCTCCCGGCGGTATAGCCGTATAGG
GCAGCCACGGGCAGACCACTAATAAAAACACAAAATTCCCATCAAGCCTCATCTTTCCATCTATTTTTAATTTTA
GTAAAAACTCCAGGAGGAGCATATATTAACTTAACTTCTCACATGACGATACTTGGCCTATAAAAACATACATTT
TCTTTTGAAGTACACCAGCTAAACTTTGGGTATATATTCTTCTTAAAACACAAACCGTGACTTACGTCTACATTC
TCTTTCAGGCCCCTTTGAAAAACTTGCCGAGGTGGCA

CtF3H1

AACTATAAAAAAGTCCCATAAAATCTATAAAAAAAAACACTTGAAAAGATCTTCGAAATCAACCACAAAAATCA
AAAAATAAAATAAAAATACTTTAAAATCAATAAAAAAAAACTTAAAAATAAAAATCCTTAGAAACCAATCAAAAA
ATTTTAAAAATCCATTGAAATTAGCCAAAAACTTTAAAAACAACATATAAGCAATGAAATTTGGTTAAAAGCATA
TCGACCAACTTTTTTTAATAGAAATGAGAACTTTTATTACCAAACCAAAACGAAACCAAGGTACAAAAACAGTT
CCTCCAGAAGTGAAAAACAATAGACACGAAACAAGATTGTCCTACAGCATATCGAACAACTTTACAGTCTTTTA
ATCCACCCACTCAGCAAAAATCATGTGTCCGTTATTAAAAGTAGTAAAGGTATAAAAAAATGATATGTCCATCTT
ATTAAAAAAATAAAAAGAAGAAATAGTAAGAAGGCAATTAACACGAGATTTATTTATTTATAAGACATCTTTAA
CTACTTTGCTAAAATAGAAAAAAAAAGTAAGGTTTTCTTCTCTAAACTTCTCCAACAACATTGCTATCTTCTTCT
CTATACTAGAAAAAACTTGAATTTTTTCAAGCCTAGAAAATTCTTTAAAATTTGATAGTAATATAGAAAAATGATT
ATTTTATTTATTCTCATCAATAAGATTTAATCTCTATCATTTTATATGTAGTTTTTGTAAGTGTATAAATATAATAAAG
TATAAAACATTATAATAGTATAGTTAAAGTTAAATATAATAAAGAATCTATTCAACTTGACTGTAAGGTCATCCTTT
ATGCCAAAGCTGATGGACACCGCTTTGGCTTTGGCTTCAACTGGTTCTCACAACACCACCATGGGTGGTTCGGT
TTCCAGCAATCCCCACCCCCTTCGGCCCCCTCTCTCTTATTTTCTTTATTAATTATTTAAAAAAATAAAAGTGTGT
AAGATTGAGTTGAATATCTTATTTTTTAGGTTAGTTTAGGTAAGGTATTAAAAAGCTGATATGGCATTGATGTAAT
AGTTGGGTTGATAGATTTAGGTTGAAACCAAATATAAAGCCATGCTTAACCAACTCTCCCAACCCCACTGCCAC
ATCAGCTTTTCTTCAACTCTTCCATTAACCCTCTGGAACCCTCTCTGATAAGTACCCAACCCACCCAACTCTCCC
CACACATAAACACACACAAAACACACACACAACAGGAGCGGTGTGACCAACCCTCCAAGGCTTCGAACCCGC
TGGTCTCGAAGCGAAACCCGCCGAACCCGCTGAGTCCGACGGGTTCGAGCCCGCTCAACCTACCCTCACGGC
CTAAAGGATGGCGAAATAATTTTTAAAGATTTTAACATTTGTTTTTCTATTTTAAAATTTTTATAACAGCGAGTTG
AAAATGTTCTTAAAAAAAGTTGACTAATTTACTCAATAAAATTATTTATGATAATCTCCCAACCCCCATTCAAAAT
AATGCATGGGGTGGTCACGTGTGGTAGTTGGAGCTCCCTTTCACCAATTTTTCAAACTCCGACCCGACCCGTTT
GTTGGAACCACCAGTACAAATAGAAGCCATTCGAAGCCTTCGAAAACGCATTCTCCCTCTCTCCATCAACAAA
CACACACACACACACACACACACACTCACACACACACACACACAAAGAACGTGCCCCAAAAAACCTATGGCT
CCGATATCGTCGTTGAAATGGGACCAGGGTTCGGCGGTCGAAAACAAGTACGTTCGCGACGAAGACGAGCGC
CCGAAGGTGCCGTACGACCAGTTCAGCAACGATATCCCGGTGATCTCGCTCGAAGGCATCGACGACGCGGACG
GTGCTGGATCCCGCAGGGGGGAGATCTGCGCGAAGATCGTGAAGGCGTGCGAGGATTGGGGGATCTTTCAGGT
CGTGGACCATGGCGTCGACACGAAGCTA

CtCHS1

TCCAAAGTCCGAGTCTGGAATTTTTTTTGAAAATCCGAGTAAAAAAAAGTCCGAGTTGCACTTTAATTCTAAAA
TCCGATACTAAATTCAAACCCAGGTCGGATTTTACCAAAATCAAACCTGAGTCGGATCTGAGTAGTAAATCAAA
ATCCGACTCTCAAAAAATTCGAAATCCGAGTCCTTTGACAATTCGAGTCGGGATTCAACTCTGATACAAATTTCA
AGTGGGAGACGACCGAAACCAAAAAATTCAATCCAAAAACTCTCAAATTCTCCAAAATCAAACCCAATCCTCA
CCAAAACTCAATCCAAATCACTTCTAAACACTTCAATCACTCCAAATCTCCATCAAATACATTCAAATTTCATGA
ATTCAACCAAAATTCTTTTCACAGACTTCATTTCTGATACAAACACAAAAACTCAAAAACTCAAAATTAAACCT
ACAAATCTATGAAATCAAACACTAAACTAATACAAACTCAATCAAAATAAAAAATCTAACACAATGAACATAAA
AACTAATTAAATCAAACACAAATCTAAAGAAATCAAAAACTCAGATCTCTAATTCAAAAAAGCTTTCATACCTC
TATTTTTCAACAAATTAGGCTCTGATACCAATTGTGAGTCTCAAAATCACAAACAGGTAGAAATAGATTTGACAA
TCAAAACTTCATAGACTTCTTGATAGAGAGATTGATATACACATGAGTTTTCTCTTCTCTATTGCCTCTCTCACA
TAATATGTTTCCTTTACAAATGAGAGGCTTGAAACCTAACAATAACCTAACATATTCTATTTATACCTATTACAATT
AAAAGGCTCAATTCTAATTAAAGCCCAACTAATTAATTAACTACAATAAAAAGCCCAACAATTAAATTAAACCTT
CGAGATTGTCTCCTTGTCTTGTGATCTCGGATACAACAAAATTCGGGTCTAACAGGATGGACGAGAACTTTCAG
CCACCTGAAGTTGGAGTTAAAACTTTTGTAAGAAATAAATAAGAAGGAATAGATGGTAAGTTGTGAAGTTTACG
TTGAGACATGAGTCCTATTTAAACATATTAATATTGATTTGGGTGTATCTTAAATTACTATAGTTATAATAAATGTAT

TTTTTAGTATATTTGTTTTATATAATTTAGTTTTAGTGGTTTAAAATAAATTTGTTTATGATAGTTTATAAAATAAATT
TAATTTATAGTTTAAACTTTAGAATTATAAAATAAATCTAGTTTATATTGTATATTATCATTACTATTGGAATTATAAA
ATAAACGTAGTCTTATAAGTAAGAAAAAAAGGAAAAGAAAAAAAAATCGGTCAATCAAGGCCCGGTTCAAGG
CTTGGGCTTTTTCTACATATAATAGACCCATGTATGGAACTCAAGGACCGGCCCAACCTGAGACCGGTCAAACA
AAGACCCAAAAAACCGGGTCTTTATGAGGCCCGGTCAAAGATCGTTCTGACCCGATTTTTGTCCACCTCTAACC
TCCCCTAATGGGTTGGCTTGGGGTTGGAGGGCATGAGTTGAAACTTGGGTTTGTTTTTTAAACATGTGGGATGA
ATGTGAGAACTTGTGGGTTAGAATGGATTGGAAGGGAAAAGTAATATAATGACAATAGTTTTTAATAAGTAATAT
AATGACAATAGTTTTTAATAAGTTGGGCCAAATATCGTACAAAAGTACAAAACTCGCTCTGCTAACAAAATTTTG
AGAAACGTGGCATTGATTATAATCGTTAACGCTACTAGTCCACTACCCTCTCATGAGGTAATTTGTAATGTGTAAT
GTTTGCAAATTCAACCTACCACACTCTCTTCCCCCTATATATACATTCCAACATTCAACCAACTCTATCACTTCAA
TTCACCTCTCCTACATCTTCTATAAATACCATCTTCACCAACATCCATCGGTGTTAATTAATTAAATTAAATTAACC
ACCCATGGCATCCTTAACTGATATTGCCGAGATCAGAAAAGCACAACGGGCAGAAGGTCCAGCCACCATTCTT
GCCATCGGCACTGCAACTCCCAACAATTGCATCTATCAAGCCGATTACCCTGATTACTATTTCCGAATCACCAAC
AGCGAACAC

CtCHS3

TTTTCAAAATTTTTGTTGATTTCAAGAGATTTTTTAAGTTAACTTTTGTTGATTTTGATGAACTTTTTTGATTTTTT
TGTTGAATCGAGAGAATTTTTTGGTTTTTTATTTGATTTTTAAAAAATCCGTTTTTTAGTTGATTTGAGAGAAATT
TTGGGTTTTCTGATTGATTTCGAAAGAATTTTATGGTTGATTGCGAGAAAAAAAAATGGGATTTTTGGTTGATTT
TGAGAGAAATTTTAGGTTTTTTAGATGATTTTGAAAGATTTTTTCCCTTTTGGTGCACCTCCTTAAGTTTTGAAA
ATAATACGATGACACCTCCCAAAATAATACGGTTGCACCTTTACAAAATTAAATTTTTTACCTCTCGTTCGATAAA
AAATACGGTGGCATGTGCCACCGTAAGCCCTTCATGAGCTCACTCCCTCTGCTCATAGTTTAATTAAGTTACAGT
TAGGGTTTATTTTTCTACAAAGAGATTAATATGACATTTATGATTTTAAATTAGCCTATAAATTGAATAATCATTAAT
TTTTATAATTTTTATAGTTTGAAAGTATAACTTGATATATTCTTTTTATGAACTAGATGTAACTTTTGTTAAACCTTT
CAAGGAGACCTTTGTCTTTTTTTTTTTTTGTAAAATTTGGTACAATTGGTACCAATACTACACCTAAAAAGCTAA
AATAATATTTCACTTATATATTTGGGTTTTTAAGTCCTCTAATATAAATCTTATTTAACACTTTAAAGTACTTGATTC
TGCTTATGGAAATTGGTACAATTGGTCCTTCAAAGAAAACCTTTAAAGCTTAATATTAATGCTTCTCCTTTAATTG
CTACCTTTGGGTGACTATTTAACACTTACCGCTGTCTGGTACTTTTGTTTGTTCTGATAAATTATGAAAAGGAAG
TTAACGATATAAAAACGCCAGGGTCCTTCTCAAGGCATTTGTAATTTGAGCTTCATGCCAGACATGGACACGTG
CAAGTAACTTGAAAATCAAGGAAATAATGGTTATGTTTGGCAAACTAGGCTGATAAAATAAATATAATCTGATAA
ACCAGTCACGTTTTATTAAAAAAAAAAAACTACCTTAGAAGTTATACGACAATGCAAAATGATATTAAAGGACA
TGTCGAGAAACTAATTGGAAAGTAAAATGGCATAGTCGGACGAAATAAAAAAGGTCTTTTTGGAACACTATCTC
AGCTAACTTTTTAAAAAGTTAGTTTATAGATGGTTTATGAAACGTGACTTTTTAAATAAAAAGAAAAAGAAAAG
GCTAATTTATGGCTTCCGAACATAGTTGATGCCTTTCAACTGTGAGATTAAACGCTATTGTAAGATAAAACGACC
CAATTTTTAGTTTAGTCATGTGTGTTATGTACACAAGCCTAGAACGATCTACACAATAAACTACAAAAAATGTAC
CTGTTTTGCGAGTCCCCATTAAAGAAATCTAATTTGTTGGATCGTCTCGGAAAGCATGCTAACAGATCTCGACAT
GAAGCATGAAGCGAAGATTTAACTTGTTTGCATAATTTATCAAACAAATTCCCGATTAAAAAATGTTTGCATTGT
TAGTGGGTCGTTTGTAACCAAAAGTCATAGCTCAGCTCTAAGAGTATCATACGAGAGATAAATTAGGTACACGG
AGGTCCGTTTGATGATTGGCTAAGATCCCGATACCCACTATCATCCAATCTCCATAGTAATGGTAACTACCACAA
ACAGATTTCCAAGTCGAAATATACTACACCGGAACCAATTGCACTACATGTCGTTAAGACGTTAACTGCTTATCT
GTGTATGTAACGGTTCTAGATTAAGAAAGGTTTATGAAAATAAATAGATACATGAATGCATCTTTAGGATAAAAA
GCTTAAAAAAAACTTATAAACGGTGCAATTTATTGAAACATTAACAACCACCGATTGTTATGACTCTAGCTACC
ATTTCTTGTCTATATATACAATGGACAAAAACCTTTTTCATATCACTTGTAACACTTGTAAAGTACTTGGAATCAA
ACAAACCGATCATC

CtF3H4

TCGAACCAGATCAAAGAAAATTGAACCAAAAATCTAGTTTAGAGAGAGAGAGCCCGCCACCCACCACTACATC
GCCGTCGCCGCAGGAAACCCCCAATTGGTTGCAATCAGATTTGTGTATGTGTGTGTTTGTGACCCACATCTGTG
TATGTGTGTGCGTGAATAGAAGAAGTGGTGTTGGTTAGAAGATGGTGGTTTGGAGATGGTGGTGGTGGTTTGCC
GACAGGGGCGATCAGAGGTGGTGGGTACAGATGAAGATGAATAGAGAGAGAGAGAGAGATAGTTACCATTTG
ACCCTTCTTCTTTCATTTAATCAAAGGCACCCCTCATTTAACTAAGTTATTAACGGGGTTAGTGAACAAGGACTA
GATCCAAAGCAAAACATGAACCCTAGGGACGTTCAGTGTACTTTTTCAAGTTTGGGGATCAATTTCGTTGGTTT
ATATAAACCTTAGGGACCAAATTTATAGTTTACTCATAATAAATGTATTATGGTATTGGCATTTAATAAACGGCGGC
GATCTTTACACCATATAATTTAATATGAAAAATAATGGTCATGAAAAAAGATTTTCATACATATATATATATATATAG
AGAGAGAAAGGTTCAAATGAGAAGTGCTAAAAAAATGCGAAGTAGAGAAGTGATCTCCACCACTCATACGGT
CAATAAATGAGGCTTTGATCGGTCCACCACTCATACGGTGGACGTGATCACGGAGGATCTGTCTCTTATACACAT
CTAGATGTGTATAAGAGACAGCCGTATGAGTGGTGGAGATCACTTCTCTACTTCGCATTTTTTTAGCACTTCTCA
TTTGAACCTAATACTATATATATACCTGTCTCTTATACACATCTAGATGTGTATAAGAGACAGCCGTATGAGTGGT
GGAGATCACTTCTCTACTTCGCATTTTTTTAGCACTTCTCATTTGAACCTAATACTATATATATACATACATATATAT
ATATATATTTATACCGAAAAACTGAGTAACCACCAAATGCATTTGTGCATACGGGTAATTTGTAATATGCACACAT
GCATAAGGAACACCTTCGTAGCCATTCATTTGGACTGTATGTATTTGAATATAAAAGTAATTTTTAAAAAATTGTA
ATATGCAGGTGTGCAAAAGAAACACCTTTGTAGTCCTTTATTTGGACCGTATGCACTTGTACATAGAAATAATTT
GTAATATGTAAATGTCCATAGTCCTTAAGTGCATAATTATAAGTACATAGATACAATCTACATAAAAGGCTATAAA
GGTATTTCTTAATTTTTTTTCTTATAGAGAGAGGAAGAGAGTGTGTGTATAGTTCACACAAAACCGAAAAAGAA
TAAAAAAAATGAGAAATACCCTAAATAACCTTTCATTTATACAATTACATATAATTAAATAATTGAACATGGTTCC
GTTCGGGTTAGCAGGTTGGCGGGTCAAAAACCTCAACCCATGACATGACCCTTTTAGCTAATCGTGTCGAGATC
TTCGACCCTTACCCGTGATGGGTCGTGTCACGGGTTGGCGAGTCAGAAACGGGTTGCTGACGGCGACGACGAC
TGGAAGAATGGAAGGCATGAGCGAGAGAGACGATTGGCATTTGGAACGGAAAGTGAGATTGAATGTCGCCTG
CTTATTGAATATTTTAGGTTACTTAGGGTTATTCACTATATTAAAAATATATAAAAAATAAATAAGTAAAAGTTAAA
CGAGTTCACGGGTGAACCCGTCAAACCCGGCGGGTCAACCCGAACACGACTCGCGAAATTGACGAGTTGGTG
GGTTCGCGGGTCAAAAACCTCAACTCAAACCCGTTTATCGTCGTGTCGTGGCGTATCCGGATGGCGGGTCGTG
CCGGAAATTGCCACCCCTAATATCATACATATAAATGATTACCAACGCGTTCCTTAGTTTCCTCTTTTTCTACATT
TCCCTACCTAACCCCTCTCTCCCTATATATATGCATATGATTGTGTATCTACACTTAAGAAAGATAGCAAAACCAC
AACCCAAACATCAAA

CtC4H2

GATTTGGATATTGATTGCGTACGGGGACAAGGGTATGATAATGGGCTAATATGAAGGAAAGCATCAAG
GTGTTCAAAGAAGAGATTACTTGATATAAATCCTAGAGCTTTTTACATGCCATGTGGATGCCATTGTCTT
AATTTGGTTTTGTGTGATATGACAAACTCTTGTCATAAAGCAAAAACTTTTTTTGGTACGTGTCAAACGA
TTTATACCGTGTTCTCTAATTCTACAAACGATTGAGTGTTTTACTTGAATATGTTGATGATTTGACTTTAA
AGTCTTTATGTACTACTTGTTGGGAAAGTCATATTGAAAGTGTCAAAGCAATAAAATCTCAACTTGGCCA
AATAAAATATGCTTTGATAAAATTAGTCCATGTATGTGAAGATGGAAAAGTGTGTAGAGATGCCGAATC
CCTGATAAATGGTGTACTTTCAAGTTTTGAGTTCGTTTTAAGTTTGGTTATTTGGCATGAAATTTTATATC
GGATTAACTTGGTTAGTAAAAAGTTAAAATCGAAAGATATGCTTCTAGATGTTGTGGTAAAAAACTTGG
AAGGATTGGTTGTTTATTTTGAAAAATACAGGGATAATGGGTTTGACCTAGCTATTATTGAGGCTAAAG
AAATTGCGGAAAACATTGGAGTTGAACCCGATTTTCCTGTAAAACGCCATGATTGTAGGAAAAAAACAT
TTTGATGAGATCCCAAATAGTGAAAGAGAACAACAATCTGCAAAAGAATCTTTTAGGGTCGATTACTTT
CTAGTTTTAGTGGATATGAAACTTTCTTAGTTAAGAAGTAGATTTGAACACATGAAACATTTCGAGTCTA
TATTTGGGTTCTTGTTTGATGCATCATAGTTGGTTAAATTGCAAGATAATGAATTAAAGAAATGTTGTTT
GAATCTTGAGGTTGCTTTGATACATGGTGACGGTGTTGATATTAAAGGAAAATATCTCTTTACCGAGTTA

CAAATTTTGCAGGGCATGATACCATCTGAAGCATACGAGGAAGAAAAGCCTTGGACGTCTATTCAAGTT
ATGGAGTTTGTAAGGAAGGTCGATATGTTCCCGCACATGTTGCTTGACTACAAGATTTTGTTAACCATAC
CGGTCACGGTAGCATCCGACGAACGAAGTTTCTCAAATTTAAAGCTTTTGAAGTCATATTTGCGGACTAC
CATGAGTCAAGAGAGGCTTAACGGGTTAGCAATTTTGAGCATCGAAAGTAAGTTTCTAACGAATGTTGA
GTATGATAGATTAATTGAAGATTTTGCTTCACGAAATGATCGAAGACATCGTTTTAGGTGATATTATGTT
TGCTTTTGTTGTTCAGGGTTTTGGTTTATGTCTTCCCCATTGTAACTTTTTTTAATAACCAACGTGGGGAT
GAGTCGCCTAGATTGACTAGGTTCCAAACCCCAAAAAAAAATTGTGTTTTTGGTTCATTAAATTAGGGGC
ATATTTTTGGTTCTCTGCCTAGGGCATCCAAAACCATTGGACCGGCCCTGACCATAAGCTAGTTTTTTTG
AAAATTTACTTTGAGTAGCGTTTTGGTGACTTATAGTAATTATGAGACTTTTTAATTTTTTAAATTACAAA
TATACCCTTCTACTTTTTACATTACTAATTTATAAACATGTCTTTTTTTATCATTTTACAGTCATAAGCTAC
CTTATCAGCTAATTTTTGCCAAACGCATCTTATTCATCAGCTACCTTATCAGCTAATTTGTCAAACATAGC
CTTAGTTAGTTTGTCAAGAATAATATCGTAAAAATAAAATACTAATATAATAACCATTTTTTCTATTATA
AAATATATATATATTTTGAAATAGAAAAAATTACATAAAATAAGTTTGCATCCAACTTTGATGCTAACTT
CTTCTGATTCAACTAACGGCGTTAGCTATGCATAGTCCCACCAACTTCACGTCCACCTAACCCCCACGTC
GCTACCTGTATAAAATCCCTACCCCCACCATTTCTTAAAATTacaaatctctcctcctctttggcaactccaaccaccaccaccatTA
TCAACCGCATGGATCTTCTCTCTTGGAGAAAGCTCTCGTAGCTCTCTTCGTCGCCATTCTAGGCGCAATC
CTCATCTCCAAGCTTCGCGGCAAGCGTTTCAAGCTCCCGCCGGGACCGATTCCGGTTCCGATTTTCGGAA
ACTGGCTCCAAGTCGGCGATGATCTCAACCACCGCAACTTAACAGATCTCGCCAAGAAGTTCGGCCAGA
TCCTGCTCCTCCGC

CtC3H1

ATGGCCTTTAACGTGGCGGCGATGCCACGCCTCGACGTTATGGCGCGTTATGGCGCCGCCATAACGCGT
TTTTTAGAACCTTGAAAAAAACACATCAACACCATTAAAACCACCATAAAAAACCCACCTCCAACCCAC
TAAAAACCGCATTAAAAACACCTTCTATATTATAATAATAATAGACATTTTATCTATTAAACTTTTAATC
AATAAGTATATATTTAATTATAAAAATTGAATGTTATAATAGTTTGCATTTTTTTTACTTTGGCCTCTAAG
ACTATGCGTACCCGAACCCTCCCAACCCCACTACCACATCAGCTTTTTATAAACTCACAATAAGCCCTTT
GGAACCCTCTCTGATCAGTACCCGTACCCTCCCAACCCTCTCACTTTTTAATAAAACACAACACACACTC
CCACACACACACCACACACACAACACACACACACACACAGCCACACACAACAAAGCAGCGGTTTAGAG
CGGTGTGGCCACACCGCTATGCCTGCAGCCCGCCGAGCCCGCTGGTGGCGTACCAGAGGCCGCTCCAGC
CCGCTAGCCCGCCGGCTTCCAACCCGCCGAGCCCAGCCCTCCCGGTACGCACGACCTAAATATAAAGGC
GTATCTTTTTGACCTCTAAACCTTTTTCGCGTTTTAGTATTGGATCGGAGATGAAAACATCACCATTTTTA
TGTTTTTAGTAGTGGGTTGGAGAAAAAACATCACCTTTTTTATGTTTTTAGTAGTAGGTTGGAGATAAAA
AATCATCATTTTTATGTTTTTAATAGTGGGTTGGATATGATCCAAGACCCTTAATTTTTTCTATTTACGAT
AGTCAGCTTAGAGGTTATTATAACATGTGTACCAAGCGTCTCCATAATAAATCCTACCGTATAAAACAAT
AAGACAAGTTGTGAAAAAAATATTTCACTACTAAAAAACTATATATTACCGACCGTTAAAAGCAGTCGG
AATTTACCAATTGCTTTTCGACGTTTACCGATTGTTCCTCTTTTTATCTTATTTCTTAATTAGTCGTCGAAA
AACGGTTTGAAAATCGTCGAAAAACGATCGATAAATGGAACAGTCAAAAAATCGTAGTTAAATACCGA
CGAAAACCATCGCCGGCTTTTAACTACCAACTTTTTATCGACGACCGTTTTTATCGAAAACACCATCGAT
AAAGCTTATTTCCGACTATTTCAATCGTCGATAATTCCCTTATCTTTTAGTAGCATTTGTCTGGTAAACGT
TTAACTGGTATTTATTCTATCAAAAACACATCCTTAGATTGTTTTCAAAAATCCCAGGGGCTCATAGGTA
GTGGAAACTTGAAACCGCCAAACCTTATAGTTTTATTCTTACATTATCCATCAACCAAACCCAACAACCA
ACCTTTTTACCAAACTACCCTTCACCAAACTCCAGCAGCTCCTAACTGAAATTACAAAACTACCCTTCAC
CAACTCCCTTCTCCCCTCTTTTCTTATATATCAACAAACATTTCCATTACTATCCACCATCCTCACCAACC
ATGGCCCTCCTACTAACGTACGCACTCCCCCTCTCCTTCACCTTCATCCTGCTAACCTACGCCCTCTACCA
CCGCCTCCGGTTCAGGCTCCCGCCGGGCCCCCGCCCGTGGCCGCTCGTCGGAAACCTCTACGACGTCAA
GCCCGTCCGGTTCCGCTGCTACGCCGACTGGGCCCACCGCTACGGCCCGATCATCTCCGTCTGGTTCGGA
TCCATCCTAAACGTCGTCGTCTCCAACAGCGACCTGGCGAGGGAGGTCCTCAAGGAGAAGGACCAGCAG
CTGGCGGACCGCCACCGGAGCCGGTCGGCGGCGAAGTTCAGCCGCGACGGCCAGGATCTGATCTGGGC
GGACTACGGGCCGCACTATGTGAAGGTCCGGAAGGTGTGCACGCTTGAGCTGTTTTCGCCCAAGAGGCT
GGAGGCCCTAAGGCCGATTAGGGAAGATGAGGTCTCGGCTATGGTGGAATCGATCTTCAAGGACTGTGT
GGATTCTGATAAAAATGGCAAGAGTCTGCTGGTTAAAAGCTATCTAGGAGCAGTGGCATTCAACAACAT
CACCCGGCTCGCTTTCGGGAAACGGTTTGTCAACTCGGAGGGCGTG
