# Supplementary material for: CtMYB1 regulates flavonoid biosynthesis in safflower flower by binding the CAACCA elements
Source: PLoS One. 2025 Dec 10;20(12):e0337921. doi: 10.1371/journal.pone.0337921 (PMC12694881; doi:10.1371/journal.pone.0337921)
Supplement: S5 Table — (PDF) [file pone.0337921.s014.pdf]

S5 Table . MYB element and synthetic sequence

MYB element sequences	Synthesized single - stranded chain (with biotin labeling)
**TTAGGTT**	AGTTAGGTTAAAGTTAGGTTAAAGTTAGGTTAA
**CAGTTG**	TCTCAGTTGAAATCTCAGTTGAAATCTCAGTTGAAA
**TGGTTA**	TTTTGGTTACAATTTTGGTTACAATTTTGGTTACAA
**TAACCA**	ACATAACCATTAACATAACCATTAACATAACCATTA
**CTGTTG**	TCACTGTTGGTGTCACTGTTGGTGTCACTGTTGGTG
**CAACCA**	TCTCAACCACCGTCTCAACCACCGTCTCAACCACCG
**TGGTTG**	CGGTGGTTGTTACGGTGGTTGTTACGGTGGTTGTTA
**TAACTG**	CCCTAACTGTAACCCTAACTGTAACCCTAACTGTAA
**CAGTTA**	AAGCAGTTAACGAAGCAGTTAACGAAGCAGTTAACG
**CACATG**	ATACACATGCGTATACACATGCGTATACACATGCGT
**CAATTG**	GTGCAATTGGTTGTGCAATTGGTTGTGCAATTGGTT
**AACCTAA**	TGAACCTAATATGAACCTAATATGAACCTAATA
**CAACAG**	CTCCAACAGCGACTCCAACAGCGACTCCAACAGCGA
**CATGTG**	CGTCATGTGAGACGTCATGTGAGACGTCATGTGAGA
**CATTTG**	TCTCATTTGAACTCTCATTTGAACTCTCATTTGAAC
**TAAGAGA**	TATAAGAGACATATAAGAGACATATAAGAGACA
